# Design Rules for Selective Binding of Nuclear Localization Signals to Minor Site of Importin α

**DOI:** 10.1371/journal.pone.0091025

**Published:** 2014-03-07

**Authors:** Xiaodong Pang, Huan-Xiang Zhou

**Affiliations:** 1 Department of Physics, Florida State University, Tallahassee, Florida, United States of America; 2 Institute of Molecular Biophysics, Florida State University, Tallahassee, Florida, United States of America; University of South Florida, United States of America

## Abstract

Selectivity is a critical issue in molecular recognition. However, design rules that underlie selectivity are often not well understood. Here, we studied five classical nuclear localization signals (NLSs) that contain the motif KRx(W/F/Y)xxAF and selectively bind to the minor site of importin α. The selectivity for the minor site is dissected by building structural models for the NLS-importin α complexes and analyzing the positive design and negative design in the NLSs. In our models, the KR residues of the motif occupy the P1’ and P2’ pockets of importin α, respectively, forming hydrogen-bonding and cation-π interactions. The aromatic residue at the P4’ position plays dual roles in the selectivity for the minor site: by forming π-stacking with W357 of importin α to reinforce the minor-site binding; and by clashing with the P5 pocket in the major binding site. The F residue at the P8’ position occupies a deep pocket, providing additional stabilization. The P7’ position sits on a saddle next to the P8’ pocket and hence requires a small residue; the A residue fulfills this requirement. The principal ideas behind these blind predictions turn out to be correct in an evaluation against subsequently available X-ray structures for the NLS-importin α complexes, but some details are incorrect. These results illustrate that the selectivity for the minor site can be achieved via a variety of design rules.

## Introduction

Molecular recognition is a major theme in biology. Proper recognition requires designing in selectivity. However, the design rules underlying selectivity are often not well understood. In eukaryotic cells, importins mediate nuclear protein import. The classical import pathway involves importin α and importin β; the former recognizes, typically via its major site, nuclear localization signals (NLSs) in cargo proteins [1]. Here we describe a set of design rules that explain why five NLSs selectively bind to the minor site of importin α.

Importin α has two functional domains: a small N-terminal domain for importin β binding (IBB) and for autoinhibition and cargo release [2]–[4]; and a large C-terminal domain for NLS binding [3], [5]. In the cytoplasm, binding of the IBB domain to importin β relieves importin α from the autoinhibited state, allowing the C-terminal domain to bind the NLS of a cargo protein. This ternary complex is then delivered to the nucleus after association with other cofactors including RanGDP and nuclear transport factor 2.

**Figure 1:**
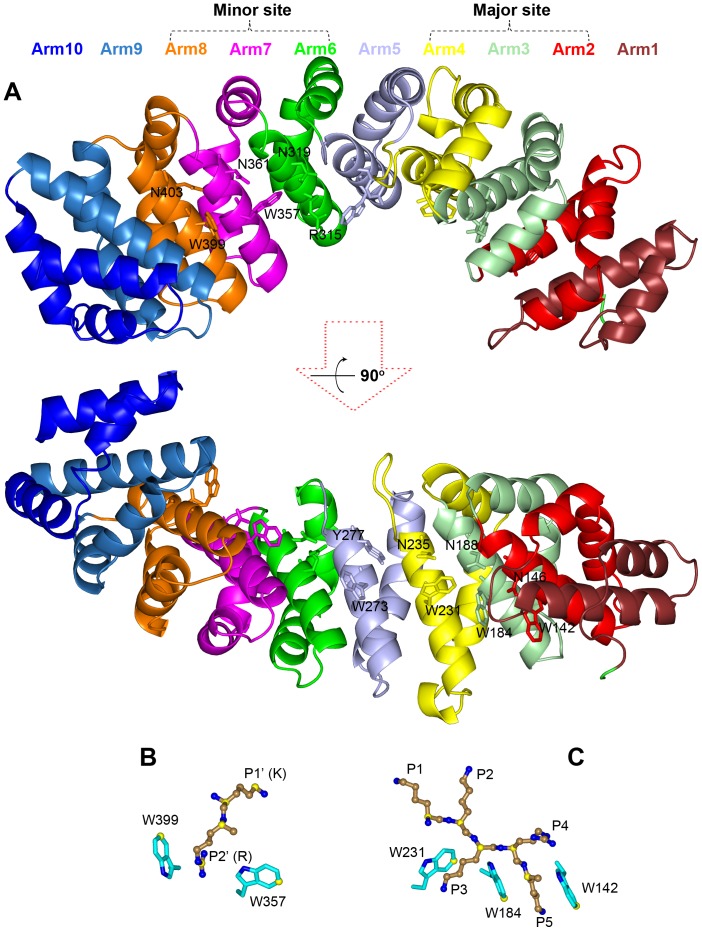
Structure of an importin α-NLS complex (PDB entry 1Q1T). (A) The 10 Arm repeats of the importin α NLS-binding domain, shown in different colors. The conversed W-N residues are shown as sticks. (B) and (C): the highly conserved importin α-NLS interactions at the minor and major sites, respectively. Importin α residues are shown as sticks with carbon atoms in cyan, and NLS residues are shown as ball-and-stick with carbons atoms in sand. Carbon atoms used for calculating positional dispersions at the minor site in 24 crystal structures and at the major site in 35 crystal structures are shown in yellow. All structure figures were generated by Pymol (http://www.pymol.org/).

As shown in Figure 1A, the NLS-binding domain of importin α (residues 72 to 497 in the mouse protein) is shaped like a twisted banana, consisting of 10 armadillo (Arm) repeats. Each repeat is composed of three helixes, H1, H2 and H3. The H3 helices from the 10 repeats form the concave surface, while the H1 and H2 helices form the convex surface. Conserved W and N residues on H3 helices of Arm2–Arm4 and Arm7–Arm8 line two separate NLS-binding sites, termed larger and smaller initially [5] and major and minor now [6].

NLSs are characterized by a single cluster of basic residues (monopartite) or two clusters of basic residues typically separated by 10–14 residue linkers (bipartite) [1]. In crystal structures (see Table 1), many monopartite NLSs are found in the major binding site. In other cases, two copies of the same NLS are bound, one at the major site and one at the minor site; effects of mutations disrupting either the major or minor site, along with other evidence, suggest preferences of these NLSs for the major binding site [5], [7], [8]. In contrast, a single copy of a bipartite NLS has the N-terminal basic cluster bound in the minor site and the C-terminal basic cluster bound in the major site (running antiparallel to the direction of importin α). At the major site, the critical residues in NLSs have been termed P1–P5 with a strictly conserved K residue at the P2 position, while at the minor site the critical residues have been termed P1’–P2’ [5], [9], [10]. For mouse importin α, the pocket formed by residues W399 and W357 at the minor site is defined as P2’, the NLS residue *i* occupying this pocket is called the P2’ residue (Figure 1B). The preceding residue *i* –1 and following residues *i* +1, *i* +2, *i* +3, … are then P1’, P3’, P4’, P5’, …, respectively. At major site, the pocket formed by residues W231 and W184 is defined as P3, the corresponding NLS residue *i* occupying this pocket is called the P3 residue (Figure 1C). The preceding residues *i* –1 and *i* –2 and following residues *i* +1, *i* +2, … are then P1, P2, P4, P5, …, respectively.

**Table 1:** Collection of Protein Data Bank entries of peptide-importin α complexes.

PDB	Peptide/Protein	P1’P2’P3’P4’P5’	P1 P2 P3 P4 P5 P6	Importin α	Reference
*Major site only*					
1Y2A	hPLSCR1		G K I S K H	Mouse	[33]
3BTR	Androgen receptor		R K L K K L	Mouse	[34]
3OQS	CLIC4		A K K Y R N	Mouse	[35]
3RZ9	Ku80		A K K L K T	Mouse	[36]
3RZX	Ku70		S K R P K V	Mouse	[36]
3TPO	IBB		L K R R N V	Mouse mut	[37]
3VE6	VEEVCP		A K K P K K	Mouse	a
4BA3	a89		G K R K Y	Mouse	[8]
4HTV	BFDV Cap		R R R R R Y	Mouse	a
*Two copies, one for each site*					
1BK6	SV40Tag	K K ? ?^b^	K K K R K V	Yeast	[5]
1EJL	SV40Tag	K K R K V	K K K R K V	Mouse	[6]
1Q1S	SV40Tag (pS_112_)	K R K V	K K K R K V	Mouse	[38]
1Q1T	SV40Tag	K R K V E	K K K R K V	Mouse	[38]
4B8O	SV40Tag	K R K V	K K K R K V	Rice	[8]
1EE4	c-Myc	K R V K L	A K R V K L	Yeast	[39]
1IQ1	IBB	K R R N V	L K R R N V	Mouse	[40]
2YNR	b54	K R K R	G K R K R H	Mouse	[8]
3L3Q	PepTM	K R R E A	K K K R R E	Mouse	[41]
*Bipartite*					
1IAL	IBB	? ?^b^	L K R R N V	Mouse	[3]
1WA5	IBB	R R R R D	A K R R N F	Yeast	[42]
1EE5	Nucleoplasmin	K R P A A	A K K K K L	Yeast	[39]
1EJY	Nucleoplasmin	K R P A A	A K K K K	Mouse	[6]
3UL1	Nucleoplasmin	K R P A A	A K K K K L	Mouse	[43]
1PJM	RB1	K R S A E	L K K L R G	Mouse	[9]
1PJN	N1N2	K R K T E	A K K S K G	Mouse	[9]
2JDQ	PB2 (Influenza)	K R D S	T K R I R M	Human α5	[44]
3FEY	NCBP1	R R R H S	H K R R K T	Human α1	[20]
3KND	TPX2	K R K H E	V K M I K T	Mouse	[29]
3TPM	MAL RPEL domain	K R K	L K R A R L	Mouse	[37]
3UKW	Bimax1	K R P L E	R K R K R V	Mouse	[43]
3UKX	Bimax2	K R K R E	K K R R R L	Mouse	[43]
3UKY	CBP80 (Yeast)	K R R G	P K R Q R I	Mouse	[43]
3UKZ	CBP80 (Mouse)	S R R R H	H K R R K T	Mouse	[43]
3UL0	CBP80 mut (Mouse)	S R R R H	H K R R K T	Mouse	[43]
3UVU	FEN1	K R K E P	K K K A K T	Mouse	[45]
*Minor site only*					
1UN0	Nup2p	M R R K I		Yeast	[14]
2C1M	Nup50	K R V A E		Mouse	[15]
2C1T	Nup2p	K R V A D		Yeast	[15]
3TJ3	Nup50	K R N A E		Human α5	[16]
2YNS	b54	K R K R H		Rice	[8]
4B8P	a89	K R K ?^b^		Rice	[8]
3Q5U	hPLSCR4	I R K W N		Mouse	[17]

a To be published.

b Not resolved.

The simultaneous use of two binding sites presumably gives bipartite NLSs higher binding affinities than NLSs that bind to the major site only. Therefore bipartite NLSs may be better able to tolerate mutations, leading to sequence diversity [11], [12]. Underscoring this point, a nonfunctional simian virus 40 large T antigen (SV40Tag) NLS with a K-to-T mutation at P2 was rendered functional through the addition of a second basic cluster residues (KR) properly positioned upstream [13]. In addition to this benefit in sequence diversity for cargo proteins, the presence of two binding sites allows for diversity in function for importin α. Besides autoinhibition through bipartite binding of the IBB sequence and loading of cargo proteins through major-site or bipartite binding of NLSs, importin α can also bind the nucleoporins Nup2p (in yeast) [14], [15] and Nup50 (in vertebrates) [15], [16] via the minor site to facilitate cargo release. Orchestrating the diverse functions would require selectivity for either binding site.

Compared to the significant efforts at the characterization of binding at the major site, monopartite binding at the minor site has received considerably less attention [7], [8], [14]–[17]. It appears harder to design sequences that selectively bind to the minor site with high affinity [7]. Two sequences identified from a random peptide library for selective binding at the minor site of a rice importin α by Kosugi et al. [7] were found to bind at either the major site only or at both the major and the minor sites of a mouse importin α, with much lower affinities [8]. However, Kosugi et al. were able to identify a class (Class 3) of peptides containing the motif KRx(W/F/Y)xxAF that selectively bound to the minor site of yeast importin α and to importin αs from rice and human, all with high affinities. Moreover, Class 3 peptides were competent for nuclear import in yeast, tobacco, and mouse cells, and each of the five identified residues in the motif was found to be important.

The present study was aimed at uncovering the design rules underlying the selectivity of the KRx(W/F/Y)xxAF motif for the minor binding site of importin α. Our approach was to build importin α-bound structural models using homology modeling, docking refinement with flexible residues, and retrained molecular dynamics (MD) simulations for five such peptides: G_1_SWAGR**KR**T**W**RD**AF**_14_; G_1_SSSHR**KR**K**F**SD**AF**_14_,; G_1_SRVQR**KR**K**W**SE**AF**_14_; G_1_SIGR**KR**G**Y**SV**AF**G_14_; G_1_SRGQ**KR**S**F**SK**AF**GQ_15_. All of these peptides were taken from Kosugi et al. [7]; the first four were screened from libraries (denoted as a58, b6, b141, and a28), whereas the last peptide is a naturally occurring NLS (denoted as GuNLS), at the C-terminus of mouse RNA helicase II/Gu. We modeled the structures of the mouse importin α-bound complexes of these peptides, hereafter referred to as NLS1–5, as part of the CAPRI exercise (http://www.ebi.ac.uk/msd-srv/capri/), which aims to make and evaluate blind structure predictions of protein complexes. Our structural models suggest that, while the KR residues anchor the peptides to the P1’ and P2’ pockets, the aromatic residue at P4’, the small A residue at P7’, and the bulky F residue at P8’ provide essential additional stabilization. Specifically, the P4’ residue forms π-stacking with W357 of importin α, the P8’ residue inserts into a deep pocket, and the P7’ residue sits on a saddle next to this pocket. When placed in the major binding site, the P4’ aromatic residue would clash with the P5 pocket. These positive and negative design rules may provide useful insights into molecular recognition.

While this paper was in revision after peer review, the structures of the importin α-bound complexes of NLS1–5 were published [18]. Hence we now have the opportunity to evaluate our structural models. The principal ideas guiding our model building, including the identification of the P1’ and P2’ residues, turn out to be correct, but some details, such as the positioning of the P7’ and P8’ residues, in which we professed less confidence, are incorrect. The lessons of our study could be instructive for structure prediction of protein-peptide complexes in general and for peptide design.

## Results

### Structural Features of Peptide-importin α Binding at the Major and Minor Sites

To identify a minimum set of structural features that defines binding at either the major site or minor site, we collected from the Protein Data Bank (PDB) 42 entries that have peptides bound to importin α at either the major site, minor site, or both (Table 1). These include 9 entries with occupancy of only the major site, 9 entries each with the two sites occupied by a copy of the same peptide, 17 entries with bipartite occupancy, and 7 entries with occupancy of only the minor site. After superimposing the importin α molecules in these entries, we found that the peptide backbones are very conserved at the P1–P5 positions of the major site and the P1’–P2’ positions of the minor site (Figure 1B,C). To quantify the geometric conservation of a particular position (e.g., P1), we calculated the distances between the locations of a representative atom (e.g., C_α_) in the superimposed structures to the centroid of these locations. Below we report the average and standard deviation of these distances. Among the 35 entries with major site occupancy, the distances of P1–P5 C_α_ atoms to their respective centroids range from 0.40±0.30 to 0.61±0.38 Å. The sidechains of three conversed W residues, W231, W184, and W142, defining the P3 and P5 pockets were also very conserved. Distances of their C_ζ3_ atoms to the corresponding centroids range from 0.35±0.29 to 0.61±0.55 Å.

Of the 9 entries that have the two binding sites each occupied by a copy of the same peptide, 7 have KR residues taking up the P1’–P2’ positions of the minor site. This is despite the fact that the KR residues take up different positions of the major site (4 entries at P2–P3 and 5 entries at P3–P4) and the likelihood that the occupation of the minor site by the peptides was accidental, forced by their high concentrations used for crystallization. Thus there appears to be a strong preference of the P1’–P2’ positions for the KR residues. Such a preference is further supported by the fact that 12 of 17 entries with bipartite peptides have KR residues at the P1’–P2’ positions, as do 5 of 7 entries with peptides bound only at the minor site. Moreover, an SV40Tag variant with a K-to-T mutation at P2 was functionally rescued through the introduction of KR residues for binding at the minor site [13]. All this mounting evidence led us to the assumption that the conserved KR residues in NLS1–5 would take up the P1’–P2’ positions.

Among the 24 PDB entries that have KR residues at the P1’–P2’ positions, the distances of P1’ K and P2’ R C_α_ atoms to their respective centroids are 0.56±0.35 and 0.58±0.35 Å, respectively, and the corresponding distances for their sidechain C_ε_ and C_ζ_ atoms are 0.48±0.26 and 0.71±0.30 Å, respectively. On the importin α side, the side chains of two conversed W residues, W399 and W357, lining the P2’ pocket have distances of 0.63±0.31 and 0.49±0.33 Å, respectively, between their C_ζ3_ atoms and the corresponding centroids.

The tendency for the occupancy of the P1’–P2’ positions by KR residues and the geometric conservation of these residues when bound to importin α form the basis of our modeling of minor-site binding. Comparing the major and minor binding sites, it appears that the latter has less capacity for conserved interactions with peptides. This difference perhaps explains the apparent difficulty in designing peptides that selectively bind to the minor site.

### Modeling NLS1–5 Binding at the Minor Site

Kosugi et al.’s discovery of Class 3 peptides as selective binders at the minor site of importin α was a remarkable feat. While these peptides were identified from random libraries, Kosugi et al. [7] noted that such an NLS (NLS5) was present at the C terminus of nucleolar RNA helicase II/Gu from mouse, and found that this NLS is necessary and sufficient for nuclear import. Using BLAST (http://blast.ncbi.nlm.nih.gov/), we found additional proteins that contain the Class 3 signature motif KRx(W/F/Y)xxAF from the non-redundant protein sequence database (Table S1). The intention of our search was not to find as many proteins that contain this motif as possible, but to find evidence that diverse proteins could use this motif for nuclear import. In line with this contention, the proteins listed in Table S1 are all localized in the nucleus. Their biological functions include DNA binding, binding to ubiquitinated histones at DNA lesion sites, and methyl transfer.

To uncover the design rules underlying the selectivity of the Class 3 peptides for the minor binding site and specifically the roles of the five conserved residues shown by Kosugi et al. [7] in mutational studies as important for importin α binding, we built structural models for five such peptides, NLS1–5, bound to mouse importin α. We generated initial models by homology modeling, refined these models by Rosetta FlexPepDock [19], and further refined the sidechains by backbone-restrained MD simulations (see Methods for more details).

**Figure 2:**
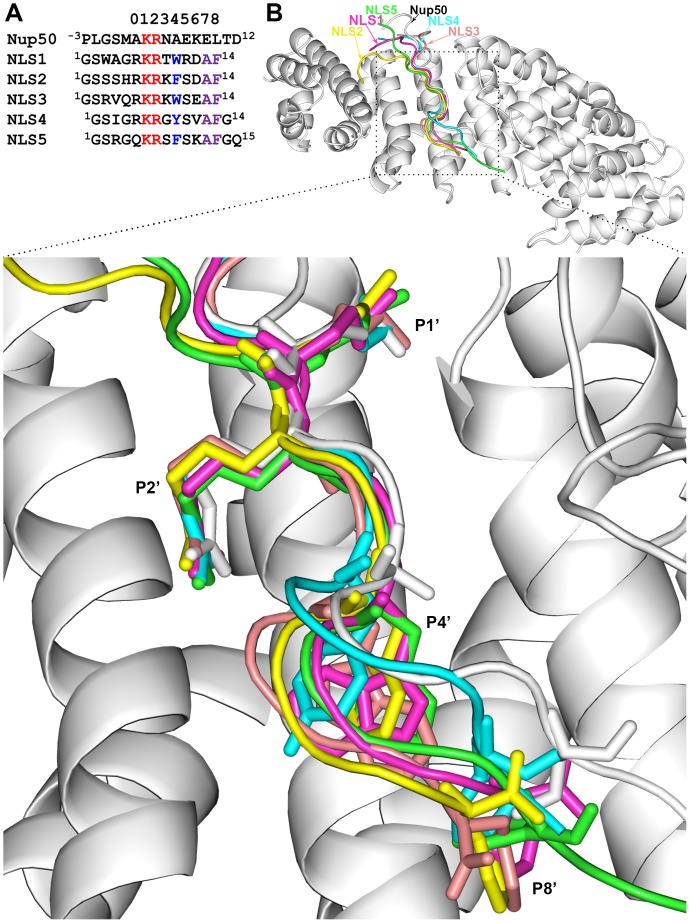
Sequence alignments of NLS1–5 with Nup50 and structures of modeled complexes with importin α. (A) Sequence alignments. The numbers at the top represent P’ positions. Conserved KR residues are in red, and conserved W/Y/F and AF residues in NLS1–5 are in blue and purple, respectively. (B) Overall conformations of NLS1–5, displayed on the Nup50-importin α template. A close-up view of the minor site is shown at bottom. The backbones as well as sidechains at the P1’, P2’, P4’, and P8’ positions, shown as sticks, have similar conformations among NLS1–5.

In 13 of the 24 PDB entries with KR residues at the P1’–P2’ positions, the peptides bound at the minor site have at most 5 residues after the P1’ position, and are thus too short to serve as templates for modeling NLS1–5. The peptide backbones in the remaining 11 entries largely trace a similar path along the concave groove of importin α. 3TJ3 falls in the middle of the bundle of backbone traces, and therefore we chose it as the template for our homology modeling (see Figure 2A for NLS sequence alignment). 3TJ3 is the structure for the complex of the N-terminal fragment of Nup50 with human importin α5 [16] (the latter, in our modeling, was replaced by a mouse importin α structure). The Nup50 nucleoporin and its counterpart Nup2p in yeast are implicated in cargo release [14]–[16]. The N-terminal fragment of Nup50 binds at the minor site of importin α, but also on the side of Arm9 and Arm10 (Figure S1), and mutational results suggest that binding at both sites are necessary for cargo release [15].

Figure 2B shows the overall conformations of NLS1–5 in our models for their complexes with importin α. From P0’ to P8’, the backbones of NLS1–5 closely follow the backbone of the template Nup50, and run along the H3 helix of importin α Arm7. There is some fraying at the P5’ position but close similarity at the P1’ and P2’ positions, taken up by the KR residues of NLS1–5; the distances of the Cα atoms of these residues from the Nup50 counterparts are 0.66±0.33 and 0.66±0.19 Å, respectively. The sidechain conformations of the conserved residues at P1’, P2’, P4’, P7’, and P8’ are also very similar among NLS1–5.

**Figure 3:**
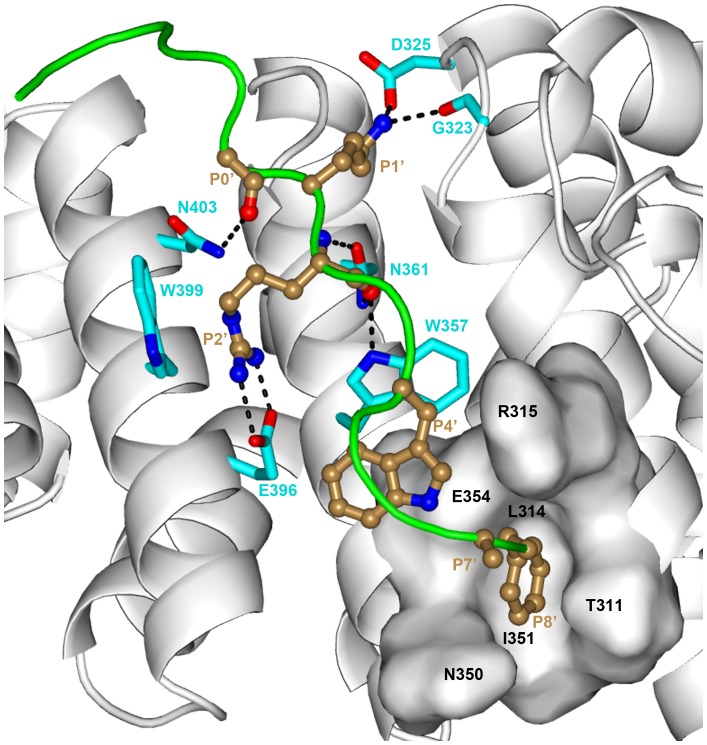
Interactions of NLS1 with the minor site of importin α. The backbones of NLS1 and importin α are shown as green tube and gray ribbon, respectively. Their key residues are shown as ball-and-stick with carbon in sand and as sticks with carbon in cyan, respectively. Hydrogen bonds are indicated with dashed lines. The binding pocket for the P8’ F residue is represented by surface.

We now use NLS1, which has the sequence G_1_SWAGR**KR**T**W**RD**AF**_14_, to describe the key interactions with importin α (Figure 3). Around the P1’ and P2’ pockets, an array of hydrogen bonds is formed between: the backbone carbonyl of P0’ and the sidechain amide of the conserved N403 on importin α Arm8; the sidechain amino of P1’ K with the sidechain carboxyl of D325 and the backbone carbonyl of G323; the guanidinium of P2’ R with the carboxyl of E396 on Arm8; the backbone amide and carbonyl of P2’ with sidechain carbonyl and amide of the conserved N361 on Arm7; and the backbone carbonyl of P2’ with the indole amine of W357. The P2’ pocket is lined on opposite sides by the conserved W357 on Arm7 and W399 on Arm8; a cation-π interaction between P2’ R and W399 provides additional stabilization. Further down the chain of NLS1, the P4’ W residue forms sandwich π–π stacking with W357. At the P8’ position, the F sidechain is deeply buried into a pocket formed by T311, L314, N350, I351, and E354. Next to the P8’ pocket, the NSL1 backbone and the R315 and N350 sidechains form a saddle, to which the small P7’ A sidechain snugly fits.

These interactions are largely conserved in the models for NLS2–5 (Figures S2–5), although there are some variations. For the P1’ K residue, in the NLS3-importin α complex, the hydrogen bonding partners switch to the sidechain hydroxyl of T328 and the backbone carbonyl of V321, along with the addition of the sidechain carbonyl of N361; in the NLS5-importin α complex, the sidechain hydroxyl of T328 joins as a hydrogen-bonding partner. For the P2’ R residue, in both the NLS3- and NLS4-importin α complexes, the sidechain hydroxyl of S360 joins as a hydrogen-bonding partner; the same also occurs in the NLS5-importin α complex, but the bidendate hydrogen bonds between P2’ R and E396 become a single hydrogen bond. In both the NLS2- and NLS4-importin α complexes, the π–π stacking between the P4’ aromatic sidechain and W357 becomes T-shaped.

### Dual Roles of the P4’ Aromatic Residue

**Figure 4:**
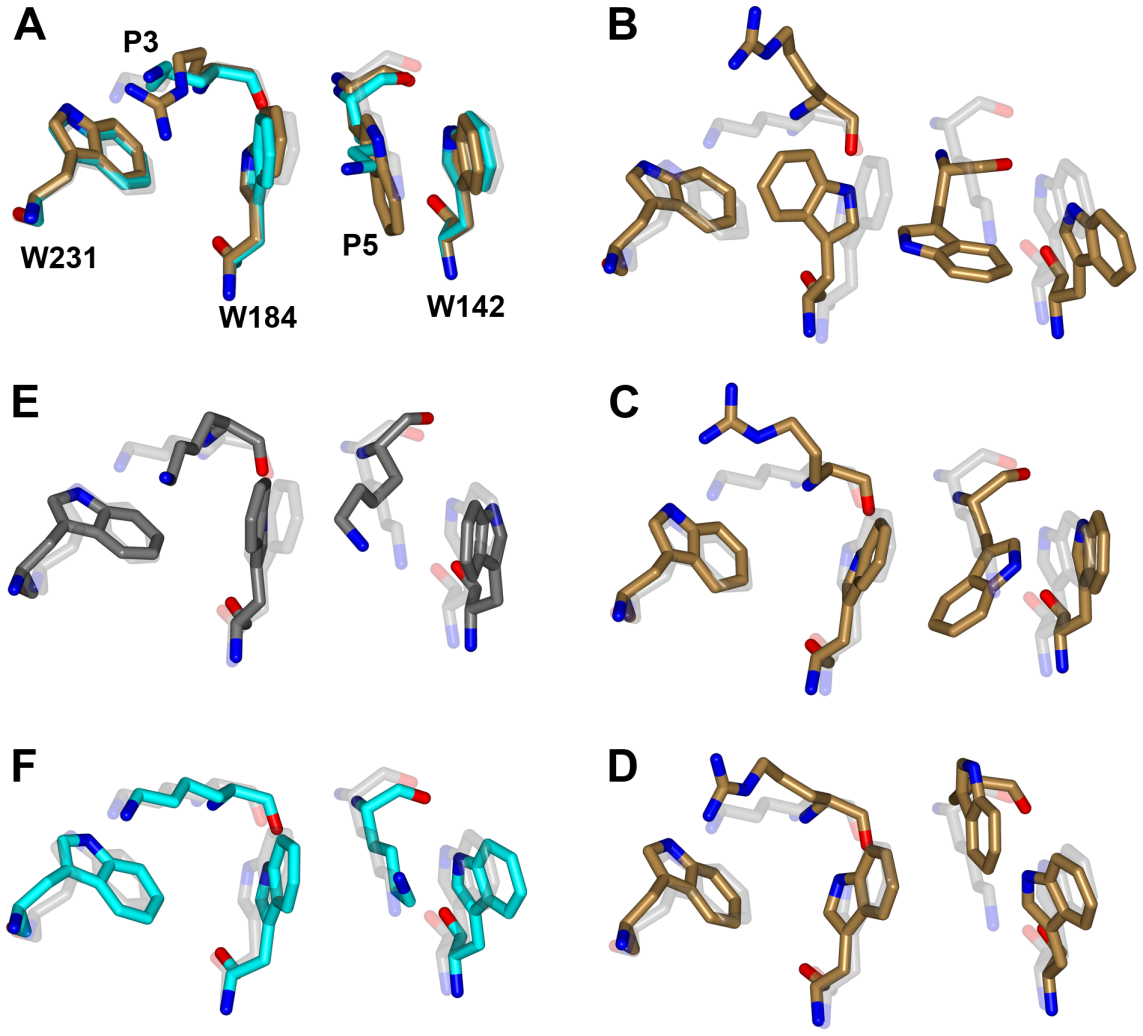
Unrestrained MD simulations of NSL3 and SV40Tag modeled into the major site of importin α. Conformations of five residues are shown to indicate whether the modeled structures are stable. Shown with carbon in light gray, cyan, and sand are the crystal (PDB entry 1EJL) and modeled structures for SV40Tag and the modeled structure for NLS3, respectively. (A) The models at the start of simulations. (B)–(D) Snapshots from three repeat simulations of the NLS3 model, at 0.85, 1.85, and 1.85 ns, respectively. (E) Snapshot from a simulation of 1EJL at 20 ns, shown in dark gray. (F) Snapshot from a simulation of the SV40Tag model at 20 ns.

To investigate why NLS1–5 select the minor site over the major site, we modeled NLS3 into the major site using 3FEY (complex of nuclear cap-binding protein 1 (NCBP1) and human importin α1 [20]) as the template. NLS3 was initially chosen because it (along with NLS2) contains four consecutive cationic residues (including the conserved KR residues), which are known to be preferred at the major site [10]. The choice of the template was not expected to have a significant effect, since, as noted above, peptide-protein interactions at the major site are highly conserved. Given that a K residue is required at the P2 position [9], there was only one choice for the alignment of NLS3 to the major site (Figure S6A), which placed the conserved KR residues at the P2 and P3 positions and the conserved aromatic residue (W in NLS3) at the P5 position. In 3FEY, P5 is taken up by a K residue. After refinement by Rosetta FlexPepDock and backbone-restrained MD simulation, the P5 W sidechain formed sandwich π–π stacking with importin α W184 and W142 on opposite sides (Figures 4A and S6B).

Is NLS3 stable while bound to the major site? To answer this question, we carried out an unrestrained MD simulation of the model. Within 1 ns, the P5 W indole rotated by about 90°, becoming perpendicular to the W142 indole; at the same time, the W184 indole also rotated by about 90°, resulting in the P3 R sidechain being pushed out of the P3 pocket (Figure 4B). Two repeat simulations using other random number seeds confirmed the instability of P5 W in the P5 pocket, although manifested in different ways. In one repeat simulation, the rotation of the P5 W was accommodated by a smaller rotation of W184 and an outward translation of W142; again, the P3 R came out of the P3 pocket (Figure 4C). In the other, both the P5 W and the P3 R came out of their respective pockets (Figure 4D).

To more fully characterize binding at the major site, we also modeled NLS1, NLS2, NLS4, and NLS5 into the major site and then carried out 20-ns long unrestrained MD simulations for all the five complexes. Further confirming the instability of binding at the major site, the bound peptides all showed large movements throughout the peptide chains (relative to the modeled structure, after superimposing the C_α_ atoms of importin α residues lining the major site). For examples, for NLS3, the C_α_ displacements were ∼1.7 Å for the P2 and P3 residues, 2.5 Å for the P4 residue, and 3.0 Å for the P5 residue. The movement of the P5 sidechain was particularly significant, with RMSD from the modeled structure at 4.4 Å, again indicating the unfavorable placement of an aromatic residue in the P5 pocket.

As control, we carried out similar unrestrained simulations of importin α with SV40Tag bound at the major site, starting from either a crystal structure (PDB 1EJL) or a model generated by the same protocol as for building NLS3 into the major site. In these simulations, lasting 20 ns, SV40Tag was stably bound at the major site (Figure 4E, F).

These unrestrained MD simulations demonstrate that, while a cationic sidechain (e.g., K in SV40Tag) can be stably bound into the P5 pocket, an aromatic sidechain like a W indole cannot. Quantum calculations have shown that cation-π stacking, compared to π–π stacking, is much more energetically favorable and results in much closer distances between the stacked rings [21]. This suggests that the spacing between W184 and W142 of importin α has been optimized for a cationic sidechain and may be too small for a W indole. We confirmed this conclusion by molecular mechanics calculations on two model systems consisting of three amino acids, i.e., a K or W residue (“guest”) stacked against two W residues (“host”) on opposite sides (similar to configurations in Fig. 4A). We obtained energy-minimized conformations of the two model systems, starting from various inter-residue distances, in explicit solvent. To compare these energy-minimized conformations, we calculated two distances between the host indoles, one for the six-membered rings and one for the five-membered rings. The sum, *d*_W-W_, is a measure for both the spacing and the parallelism between the two host indoles. With a K residue as the guest, the energy-minimized conformations have the lowest *d*_W-W_ at 14.5 Å. With a W residue as the guest, the lowest *d*_W-W_ is increased to 15.4 Å.

It thus appears that the conserved aromatic residue in NLS1–5 is partly responsible for their weaker binding at the major site. This may be viewed as a negative design. On the other hand, the favorable π-stacking formed by this residue at the minor site can be viewed as a positive design. The P4’ aromatic residue thus seems to play dual roles in the selectivity of NLS1–5 for the minor site.

### Differential Binding Stability at the Minor and Major Sites Manifested in Unrestrained MD Simulations

To gain further insight into the difference in stability between minor-site and major-site binding, we carried out unrestrained MD simulations of our minor-site bound models, in addition to the unrestrained simulations described above for the major-site bound models. Over theses simulations, each up to 20 ns, MM-PBSA [22]–[24] calculations were done to find the overall difference in binding free energy between the two sites as well as major contributions to this difference. We recognize that calculating peptide-protein binding free energy is still an extremely challenging problem, and MM-PBSA as an inexpensive, empirical method has significant uncertainties in the predicted results [25]. Nevertheless we found that overall the peptides have much more favorable binding free energies at the minor site than at the major site. For example, the binding free energies of NLS3 at the minor and major sites are −34 and −22 kcal/mol, respectively. While the two respective magnitudes have significant uncertainties due to the inherent limitation of the method, the direction of their difference is perhaps meaningful. The major contribution to the more favorable binding free energy at the minor site comes from the electrostatic component (Coulomb interaction plus solvation).

**Figure 5:**
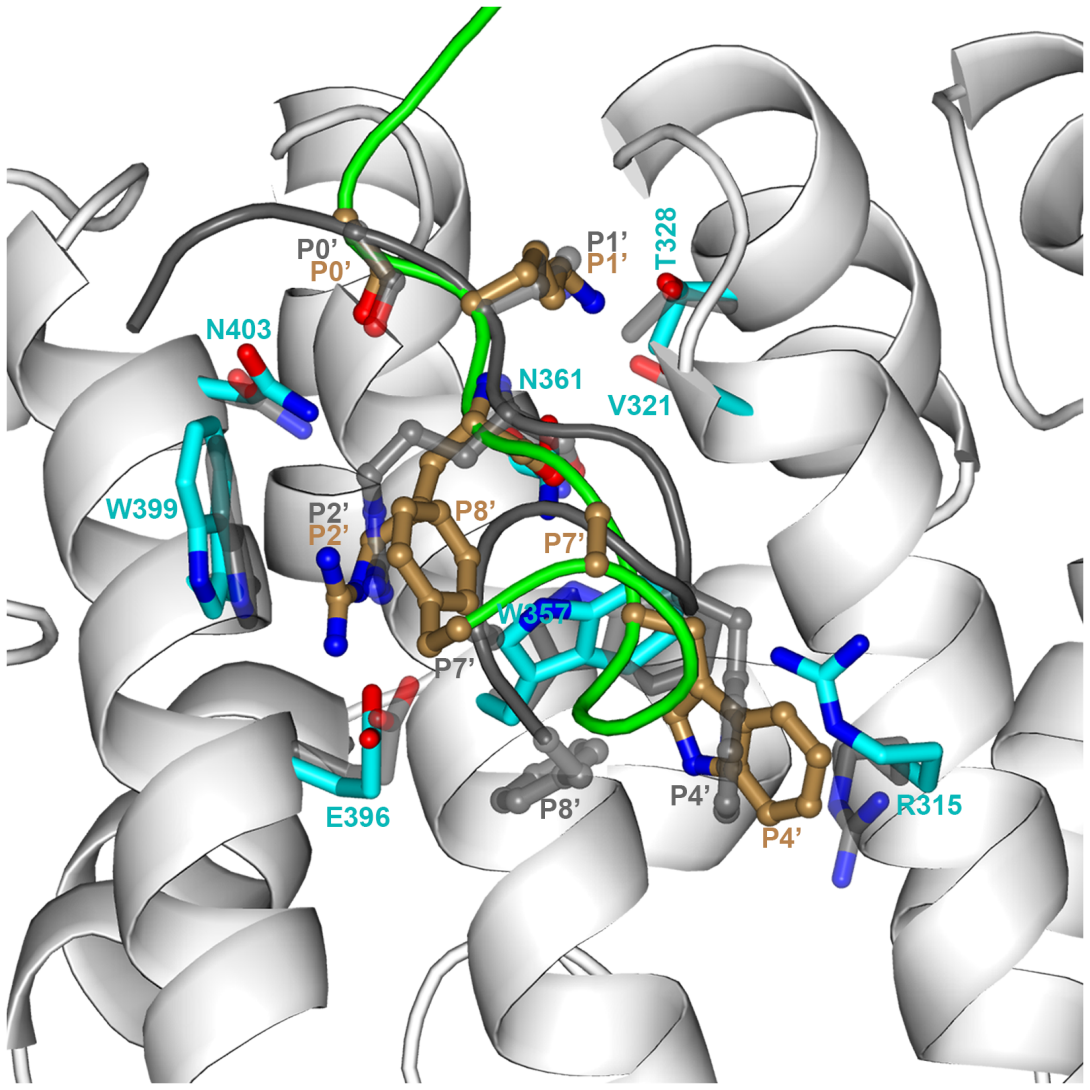
Comparison the unrestrained MD snapshot at 20-ray structure for the minor-site bound NLS3-importin α complex. The MD simulation started from our modeled structure; superposition to the X-ray structure was done on the Cα atoms of importin α residues within 5 Å of NLS3. The color scheme for the MD snapshot is the same as in Figure 3. For the X-ray structure, the backbone of importin α is undisplayed for clarity and the backbone of NLS3 is shown as dark gray tube; key residues of the peptide and protein are shown as ball-and-stick and as sticks, respectively, both with carbon in gray.

During the unrestrained MD simulations of the minor-site bound models, different parts of the peptides and the corresponding protein environments showed different extents of conformational relaxation. The P1’–P2’ KR residues were quite stable, the P4’ aromatic residue underwent moderate local rearrangement, while the P7’–P8’ AF residues experienced significant conformational change, to form a partial α-helix at the C-terminus (see Movie S1 and Figure 5 for illustration on NLS3). These differences are reflected in the displacements of NLS3 residues (relative to the modeled structure, after superimposing the C_α_ atoms of importin α residues lining the minor site; Figure S7A). In the 20-ns simulation, the C_α_ displacements stayed around 0.8 Å for the P1’–P2’ residues, reached 1.8 Å for the P4’ residue, and climbed to 10.9 Å for the P7’–P8’ residues. For the P4’ aromatic residue, instead of the π-stacking interaction with W357 as we modeled (Figure S3), a cation-π interaction with R315 was formed (Figure 5). On the protein side, the backbone of the residues lining the minor site was very stable, with a C_α_ root-mean-square-deviation (RMSD) of 1.0 Å, but some of the sidechains showed more significant adjustments. In particular, displacements of sidechain “tip” atoms were modest (∼0.7 Å) for T328 and N361 (both interacting with P1’; see Figure 5), and N403 (interacting with P0’), moderate (∼1.5 Å) for E396, W399, and W357 (interacting with P2’), and relatively large (∼2.0 Å) for R315 (Figure S7B).

## Discussion

### Design Rules for Selectivity at Minor Binding Site

The common features in the structural models of NLS1–5 bound to importin α suggest how selectivity for the minor site can be achieved. A K residue at the P1’ position can form multiple hydrogen bonds; possible acceptors on importin α include the backbone carbonyls of V321 and G323, sidechain carboxyl of D325, sidechain hydroxyl of T328, and the sidechain carbonyl of N361. An R residue is ideally suited for the P2’ position, where it forms bidendate hydrogen bonds with E396 and cation-π interaction with the conserved W399. These interactions may explain why R is apparently strictly conserved at P2’ (Table 1); a mutation to A or even to K dramatically compromised the competence of the NLS1–5 class of peptides for nuclear import [7].

The P4’ position prefers an aromatic residue, as a mutation to V significantly reduced nuclear import activity [7]. In our model building we placed the P4’ aromatic residue next to W357 to form π-stacking interaction, but in subsequent unrestrained MD simulations the P4’ aromatic residue switched to cation-π interaction with R315. We also proposed that the P8’ F residue inserts into a deep pocket, and identified the pocket as one formed by T311, L314, N350, I351, and E354. Furthermore, we suggested that the P7’ A residue sits on a saddle next to the P8’ pocket. In subsequent unrestrained MD simulations, the backbone and the sidechains of the P7’ and P8’ residues rearranged. Our initial ideas regarding the placement of these two residues were motivated by the observations of Kosugi et al. [7] that a mutation of the P8’ F residue to A nearly abolished the nuclear import activity, and even a hydroxyl addition (by mutation to Y) significantly impaired nuclear import, as did a mutation of the P7’ A residue to V.

The critical biological functions of importin α have resulted in a large number of crystal structures for their complexes with various partner proteins and peptides, as well as numerous NLS sequences. These resources have been essential to our modeling effort. We are more confident of the placement of the P1’ and P2’ positions and less so of the P7’ and P8’ positions. We also caution that the rest of a cargo protein can potentially perturb the bound structure of the NLS. Nevertheless, as further discussed below, it appears that important lessons about design rules for selectivity at the minor site have been learned, and these might be useful for understanding molecular recognition in general.

### Evaluation of our Models against the Now Available X-ray Structures

As noted in Introduction, we carried out the structural modeling for the peptide- importin α complexes as part of the CAPRI exercise. During the revision of this paper, the X-ray structures of these complexes were published by Chang et al. [18]. Below we evaluate our models against the X-ray structures (see Table 2 and Figures S8–S13). The NLS-binding domain of importin α is known to have a very rigid backbone structure (e.g., as indicated by close superposition of 24 different importin α structures; see Methods). Not surprisingly, the importin α molecules in our models agree well with the X-ray structures, with C_α_ RMSDs <0.5 Å. Our evaluation is thus focused on the peptides.

**Table 2:** RMSDs (in Å) of modeled and X-ray structures for the NLS-importin α complexes^a^.

	Minor site					Major site		
	P1’–P2’ C_α_	P1’–P2’ sidechain	P4’ C_α_	P4’ sidechain	P7’–P8’ C_α_	P2–P6 C_α_	P2 sidechain	P5 sidechain
NLS1	0.4	1.0	1.1	4.4	9.8	0.8	1.0	2.8
NLS2	0.5	0.9	2.1	3.8	7.9	0.7	0.9	1.7
NLS3	0.4	0.6	2.9	4.9	8.4	0.8	0.8	2.7
NLS4	0.4	0.8	1.4	3.5	8.9	0.7	0.7	1.5
NLS5	0.5	1.7	1.2	2.2	8.9	0.6	1.2	1.7

a RMSDs of model were calculated after superposition to the X-ray structure using the C_α_ atoms of importin α residues within 5 Å of NLS3.

The principal ideas guiding our model building turn out to be correct. For the minor-site bound models, these include the identification of the KR residues in the Class 3 motif KRx(W/F/Y)xxAF as the P1’ and P2’ residues (Figures S8–S12). As a result, our models have very small C_α_ RMSDs (≤ 0.5 Å) from the X-ray structures for the P1’–P2’ residues. The sidechains of these residues are also close to their counterparts in the X-ray structures (sidechain RMSD at 0.6 to 1.7 Å). However, the sidechain of the P4’ residue is incorrectly placed; instead of the π-stacking interaction with W357 as we modeled, a cation-π interaction with R315 is formed in the X-ray structures. Interestingly, the latter interaction was formed during the unrestrained MD simulations, although the P4’ and R315 sidechains were still not accurately placed (Figure 5). We further proposed that the P8’ F sidechain inserts into a deep pocket, but it is clear now that the pocket is misidentified. In the X-ray structures, the peptide backbones form a partial α-helix, bringing the P8’ F sidechain back to near the P4’ aromatic sidechain and into a pocket separated by a ridge from the P2’ pocket; the P7’ A sidechain projects into the ridge, thus explaining why a small residue is required at the P7’ position. The many choices for the backbone conformations and sidechain interactions of the AF residues illustrate the challenges in structural modeling of protein-peptide complexes. That the partial α-helix at the C-termini of the peptides was formed in unrestrained MD simulations (Movie S1 and Figure 5) at least offers some hope.

Likely due to the high peptide concentrations used for crystallization, in the X-ray structures the same peptides are bound at both the minor and major sites. Chang et al. [18] confirmed the selectivity of NLS1–5 for the minor site of mouse importin α by measuring the binding affinities for the two sites. Our major-site models are in very good overall agreement with the X-ray structures (Table 2 and Figure S13). For example, the C_α_ RMSD for the P2–P6 residues is only ∼0.7 Å from the X-ray structures. The success with sidechain placement is more mixed. The sidechain RMSDs are ∼ 1 Å for the P2 residue, but larger (1.5 to 2.8 Å) for the P5 residue, once again highlighting the instability in placing an aromatic residue in this position.

### Contrasting Strategies for Achieving Selectivity at Major and Minor Sites

The major binding site of importin α involves three conserved W-N pairs (on Arm2–Arm4), whereas the minor site involves two (on Arm7–Arm8). The different numbers and arrangements of these W residues in the major and minor sites (Figure 1) contribute to the difference in selectivity between the two sites. At the major site, A K residue is strictly conserved at the P2 position [9], [10]. The three conserved W residues (W231, W184, and W142) line up to delimit the P3 and P5 pockets, suitable for long, cationic residues, i.e., K and R, for cation-π interactions. Hodel et al. [12] studied how P1–P5 alanine mutations affected the binding free energies of the SV40Tag and c-Myc NLSs, and found the effects to be most prominent at P2, intermediate at P3 and P5, and weak at P1 and P4. The major site thus seem to prefer the sequence motif K(K/R)x(K/R) for the P2–P5 positions. This motif by itself may be sufficient for major site binding [7], [12].

At the minor site, a K residue is highly preferred at the P1’ position (see Table 1), and as noted above, an R residue seems to be strictly conserved at the P2’ position, accommodated in a pocket delimited by the conserved W399 and W357. No consensus sequence has emerged for other positions at the minor site. The KR motif on its own is unlikely to yield the level of binding affinity for the minor site that the K(K/R)x(K/R) motif does for the major site, but affinities can be enhanced by additional interactions at neighboring positions. The fact that there is no consensus sequence at neighboring positions means that there is no single strategy for these affinity-enhancing interactions. In NLS1–5, these are provided by the P4’ aromatic residues and the AF residues at the P7’–P8’ positions. In Nup20 and Nup50, additional interactions are formed on the side of Arm9 and Arm10 [14]–[16] (Figure S1). Mutations on the KR motif at the P1’–P2’ positions and on two residues involved in the additional interactions suggest that binding at both sites is necessary for Nup50 to function effectively in facilitating cargo release [15].

### Positive and Negative Design Involving an Aromatic Residue

NLS1–5 contain the motif KRx(W/F/Y). This differs from the major-site motif K(K/R)x(K/R) only by the substitution of an aromatic residue by a cationic one in the last position. For NLS1–5 to selectively bind to the minor site, this aromatic residue should not be well tolerated at the P5 pocket.

Noncovalent interactions involving aromatic residues are of great importance in molecular recognition [26]. Importin α-NLS interactions provide a good example. As noted above, importin α uses five W residues to form recognition pockets at both the major and minor sites. As we proposed, in NLS1–5 the aromatic residue at the P4’ position can facilitate the binding at the minor site (e.g., by forming π-stacking with W357 of importin α), which is a form of positive design [27], [28]. On the other hand, as we demonstrated here by both unrestrained MD simulations and molecular mechanics calculations, this aromatic residue interferes with binding at the major site due to clashes with the P5 pocket, which is a form of negative design [27]. The P5 pocket favors a long, cationic residue, i.e., K or R, for cation-π stacking. Quantum calculations have shown that cation-π stacking, compared to π–π stacking, is much more energetically favorable [21].

The proposed dual roles of the P4’ aromatic residue of NLS1–5 can find support in studies of other NLSs. The human phospholipid scramblase 4 (hPLSCR4) SNL, like NLS1–5, selectively binds to the minor site of importin α, with the sequence IRKW taking up the P1’–P4’ positions and the P4’ W forming weak π-stacking with W357 (PDB 3Q5U) [17]. In 3FEY, the P4’ H sidechain (in one of two possible conformers) forms sandwich π–π stacking with W357. In 3KND (complex of the target protein for Xenopus kinesin-like protein 2 (TPX2) and mouse importin α) [29], the P4’ H sidechain forms T-shaped π stacking with W357. The TPX2 NLS is bipartite; the minor-site P1’–P4’ positions are taken up by the motif K_284_RKH_287_ and the major-site P2–P5 positions are taken up by the motif K_327_MIK_330_. Despite the three consecutive cationic residues in the first motif and a lack of a cation in the second position of the second motif, H_287_ is not able to dislodge K_330_ from the P5 pocket and opts instead for the P4’ pocket. This is reminiscent of the proposed dual roles of the P4’ aromatic residue of NLS1–5.

Further support is provided by the results on the sequence V_1_HLTVLKKRKYW_12_, identified from a random peptide library for selective binding at the minor site of a rice importin α and denoted as a89 by Kosugi et al. [7]. A later study found that, on mouse importin α, a89 binds only to the major site, with a 20-fold lower affinity [8]. In the complex with rice importin α, K_8_RK_10_ takes up the P1’–P3’ positions (PDB 4B8P). Though Y_11_ is missing in the crystal structure and so its π stacking with W357 cannot be confirmed, its assumption of the P4’ position does suggest a positive role for the minor-site binding. In the complex with mouse importin α, K_8_RKY_11_ takes up the P2–P5 positions, but R_9_ is displaced from the P3 pocket (PDB 4BA3). The displacement may explain the low affinity, and is surprisingly similar to what we found from the unrestrained MD simulations of the model in which NLS3 was built into the major site (Figure 4B–D).

### Unique Roles of the Minor Site

It has been thought that the minor site of importin α may play only an auxiliary role, in assisting the binding of the IBB sequence for autoinhibition and of bipartite NLSs for nuclear import. The fact that nucleoporins can selectively bind to the minor site to facilitate cargo release [14]–[16] suggests that the minor site may impart importin α with additional functions. The recent discovery of NLSs that selectively bind to the minor site for nuclear import [7], [17] further supports the unique roles of this site.

Bipartite NLSs derive binding affinities from interactions at both the major and minor sites. Therefore there is room for some of these interactions to be less optimal, and correspondingly deviations from consensus sequences can be tolerated, leading to sequence diversity [11], [12]. An example is the TPX2 NLS, where, owing to the minor-site binding via the motif K_284_RKH_287_, the major-site motif K_327_MIK_330_ has an M residue replacing the consensus K/R. The strong contribution of the minor-site motif is demonstrated by the observation that the minor-site fragment was as effective as the bipartite sequence in pulling down importin α from bacterial lysates, whereas the major-site fragment was ineffective [29]. A deletion of the major-site motif is an extreme form of sequence diversification, rendering the TPX2 NLS a minor-site only binder. Other classes of minor-site only binders like hPLSCR4 and NLS1–5 (see also Table S1) further expand the sequence diversity of NLSs.

A potential advantage for minor-site only binding is that it may enable these binders to avoid competition with the large population of cargo proteins that target the major site of importin α. The reduced competition at the relatively less crowded minor site therefore allows for lower binding affinities of NLSs. For example, hPLSCR4 is functional by binding selectively to the minor site with a binding affinity in the µM range, which would be too weak to compete against the generally nM binders exemplified by SV40Tag at the major site. To avoid the competition, it has to be assumed that importin α can simultaneously carry both a minor-site binder like hPLSCR4 and a major-site (or perhaps even bipartite) binder like SV40Tag as cargos. In support of this scenario, Pumroy et al. [16] has shown that Nup50, a minor-site binder, and Influenza PB2, with a bipartite NLS, can both bind to importin α to form a trimeric complex.

In conclusion, the selectivity of NLS1–5 for the minor site of importin α has been dissected here, leading to a set of design rules involving both favorable interactions in five positions at the minor site and clashes with the major site. The results highlight the unique roles of the minor site and provide new insights into molecular recognition and peptide design.

## Methods

### Overall Strategy for Model Building

Structural models for NLS1–5 bound to the minor site of mouse importin α were built by following a protocol consisting of three steps: initial model generation by homology modeling; refinement by Rosetta FlexPepDock [19], and sidechain refinement by backbone-restrained MD simulations. For investigating whether NLS1–5 can stably bind to the major site, a similar protocol was followed to build models for these peptides and SV40Tag bound to the major site. Below we present some details of the three steps.

### Initial Model Generation by Homology Modeling

We chose 3TJ3, which is the structure for the complex of human importin α5 with the N-terminal fragment of Nup50 [16], as the template for our modeling of minor site binding (see Results for the rationale leading to this choice). We manually aligned NLS1–5 to the Nup50 fragment. Because we wanted to model NLS1–5 binding to mouse importin α, we replaced the structure of human importin α5 in 3TJ3 by a structure of mouse importin α (from PDB entry 1EJL). The two importin α proteins share 47% sequence identity.

As shown in Figure S1, the N-terminal 12 residues of Nup50, plus four upstream residues (residues −3 to 0) from expression vector, run along the H3 helix of importin α Arm7, with the K3 and R4 residues defining the P1’ and P2’ positions. NLS1–5 have either 14 or 15 residues. We aligned them with the Nup50 fragment (residues −3 to 12) by lining up their KR residues and not allowing for any gaps (Figure 2A).

To replace human importin α5 in 3TJ3 by mouse importin α in 1EJL, we superimposed C_α_ atoms of the former molecule’s residues within 5 Å of the Nup50 fragment to the corresponding C_α_ atoms of the latter molecule. The resulting complex of the Nup50 fragment with mouse importin α was then used to generate initial models for NLS1–5 bound to the minor site of mouse importin α by homology modeling, using the NLS alignments of Figure 2A and the program Modeller [30]. For modeling major-site binding, the template was 3FEY and the sequence alignments are shown in Figure S6A.

### Refinement by FlexPepDock

FlexPepDock [19] is a method for peptide-protein docking and refinement, implemented within the Rosetta framework. Here we used its refinement module, which incorporates backbone flexibility for the peptide and sidechain flexibility for both the peptide and protein, to refine the initial models generated by the homology modeling. The refinement module of FlexPepDock is particularly suitable for the case where the binding site of the peptide is approximately known. We had high confidence in the binding pockets for the P1’–P2’ residues at the minor site and the P1–P5 residues at the major site. Although FlexPepDock did not allow for flexibility for the backbone of the protein, this restriction would not have much effect on the results, because structure comparison shows that the NLS-binding domain of importin α exhibits very little backbone flexibility. For example, the 24 PDB entries that have KR residues at the P1’–P2’ positions (see Table 1), spanning four species (mouse, human, rice, and yeast), all have C_α_ RMSDs <1 Å from each other.

For NLS1–5 bound to the minor site, 6,200 to 9,400 refined models were generated by FlexPepDock. Given that the positions of P1’ and P2’ residues and W399 and W357 are highly conserved in the aforementioned 24 PDB entries, we filtered out models in which these residues moved too far. Specifically, the filters consisted of maximal distances of 1.5 Å for P1’ K and P2’ R C_α_ atoms and sidechain C_ε_ and C_ζ_ atoms and 2.0 Å for W399 and W357 C_ζ3_ atoms, measured from the centroids in the 24 PDB entries. About 1% of models passed the filtering. From the remaining models, one that has the P4’ aromatic residue forming π stacking with W357 and the P8’ F residue inserted to a deep pocket was chosen for each of NLS1–5.

Similarly, for NLS3 bound to the major site, 3,000 models from FlexPepDock were filtered with distance cutoffs of 1.5 Å for P1–P5 C_α_ atoms, and 1.4 Å, 1.2 Å, and 2.2 Å, respectively, for W231, W184, and W142 C_ζ3_ atoms. The 532 remaining models were clustered according to NLS3 C_α_ RMSDs, and a representative of the largest cluster was chosen. Other major-site bound models were similarly generated.

### Sidechain Refinement by Backbone-restrained MD Simulations

Each model chosen from the FlexPepDock runs was subjected to an MD simulation to further refine the sidechains (while restraining the backbones). The simulations were performed by using NAMD [31] with the CHARM27 force field. Each model was solvated in TIP3P water with NaCl at 0.15 M. Backbone N, C_α_, and C atoms were restrained with a force constant of 10 kcal/mol/Å^2^. After energy minimization, simulations were run at constant pressure, with the periodic boundary condition. Van der Waals interactions were calculated with a switching distance of 10 Å and a cutoff of 12 Å, and updated every other step; electrostatic interactions were treated by the particle mesh Ewald method [32] with a 12 Å cutoff and updated every 4th step. Each system was gradually heated to 300 K with a temperature increment of 50 K, a simulation time of 100 ps at each temperature, and a timestep of 1 fs. After reaching 300 K, the simulations were continued at this temperature for up to 5 ns with a timestep of 2 fs.

### Relaxation in Unrestrained MD Simulations

The unrestrained MD simulation results presented in Figure 4 were obtained by continuing the simulations described in the preceding subsection (using the CHARM27 force field), except that now the restraints on the backbone atoms were removed. To calculate the relative binding free energy of the same peptide binding at the minor and major sites, we used the MM-PBSA method [22]–[24], which was available in Amber12. We thus carried out fresh unrestrained MD simulations of our modeled structures using the latter program with the ff99SB force field. To start, each protein-peptide complex was solvated in TIP3P water. The periodic boundary condition was applied. Van der Waals interactions were calculated with a cutoff of 8 Å; electrostatic interactions were treated by the particle mesh Ewald method [32] with a cutoff of 8 Å. First the system was minimized while restraining the solute atoms with a force constant of 2 kcal/mol/Å^2^, for a total of 1000 steps (500 steps of the steepest descent plus 500 steps of conjugate gradient). Then the system was heated under constant volume for 50 ps, with an increase of the temperature from 0 to 300 K, and subsequently equilibrated under constant temperature and pressure for another 50 ps, while maintaining the restraint of 2 kcal/mol/Å^2^ on the solute atoms. The equilibration was further extended for 500 ps without any restraint. Finally the unrestrained simulation was continued for 20 ns. All bonds involving hydrogen atoms were constrained to their equilibrium distance with the SHAKE algorithm, thus allowing for a time step of 2 fs.

### MM-PBSA Calculations

The python script MMPBSA.py [24] was used to calculate the binding free energies of NLS1–5 with importin α, over 2000 snapshots sampled from each 20-ns simulation. The dielectric constants for solute and solvent were 1.0 and 80.0, respectively, and the ionic strength was 0.1 M. The configurational entropy term was neglected, since this term is likely to be similar for minor-site binding and for major-site binding and our primary interest was in the difference in binding free energy between these two sites.

## Supporting Information

Figure S1 **Structure of the Nup50-importin α complex (PDB entry 3TJ3).** Importin α is shown in gray and Nup50 in green for residues −3 to 12 and red for the remainder.
(TIF)

Figure S2 **Interactions of NLS2 with the minor site of importin α.** The color scheme is the same as in Figure 3.
(TIF)

Figure S3 **Interactions of NLS3 with the minor site of importin α.** The color scheme is the same as in Figure 3.
(TIF)

Figure S4 **Interactions of NLS4 with the minor site of importin α.** The color scheme is the same as in Figure 3.
(TIF)

Figure S5 **Interactions of NLS5 with the minor site of importin α.** The color scheme is the same as in Figure 3.
(TIF)

Figure S6 **Model of NLS3 bound to the major site.** (A) Sequence alignments of NLS3 and SV40Tag to NCBP1. The numbers at the top represent P positions. (B) The model for NLS3 bound to the major site of importin α, after refinement by Rosetta FlexPepDock and backbone-restrained MD simulation. The color scheme is the same as in Figure 3.
(TIF)

Figure S7 **Relaxation of the minor-site bound model of NLS3 during an unrestrained MD simulation, as measured by RMSDs from the model.** Superposition was done on the C_α_ atoms of importin α residues within 5 Å of NLS3. (A) Cα RMSDs of different parts of the peptide. (B) Sidechain tip atom RMSDs of protein residues that interact with the peptide. Tip atoms are: C_δ_ for E; C_γ_ for N; C_ζ_ for R; O_γ1_ for T; and N_ε1_ for W.
(TIF)

Figure S8 **Comparison of the modeled and X-ray structures for the minor-site bound NLS1-importin α complex.** The color scheme is the same as in Figure 5.
(TIF)

Figure S9 **Comparison of the modeled and X-ray structures for the minor-site bound NLS2-importin α complex.** The color scheme is the same as in Figure 5.
(TIF)

Figure S10 **Comparison of the modeled and X-ray structures for the minor-site bound NLS3-importin α complex.** The color scheme is the same as in Figure 5.
(TIF)

Figure S11 **Comparison of the modeled and X-ray structures for the minor-site bound NLS4-importin α complex.** The color scheme is the same as in Figure 5.
(TIF)

Figure S12 **Comparison of the predicted and X-ray structures for the minor-site bound NLS5-importin α complex.** The color scheme is the same as in Figure 5.
(TIF)

Figure S13 **Comparison of the predicted and X-ray structures for the major-site bound NLS3-importin α complex.** The color scheme is the same as in Figure 5.
(TIF)

Table S1 **Native proteins with sequences matching NLS1–5.**
(DOCX)

Movie S1 **Relaxation of the minor-site bound model of NLS3 in a 20-ns unrestrained MD simulation.** The X-ray structure is shown as reference. For color scheme see Figure 5 legend. Movie generated by VMD (http://www.ks.uiuc.edu/Research/vmd/).
(MPG)
