# Supplementary figures and images for: Design Rules for Selective Binding of Nuclear Localization Signals to Minor Site of Importin α
Source: PLoS One. 2014 Mar 7;9(3):e91025. doi: 10.1371/journal.pone.0091025 (PMC3946659; doi:10.1371/journal.pone.0091025)

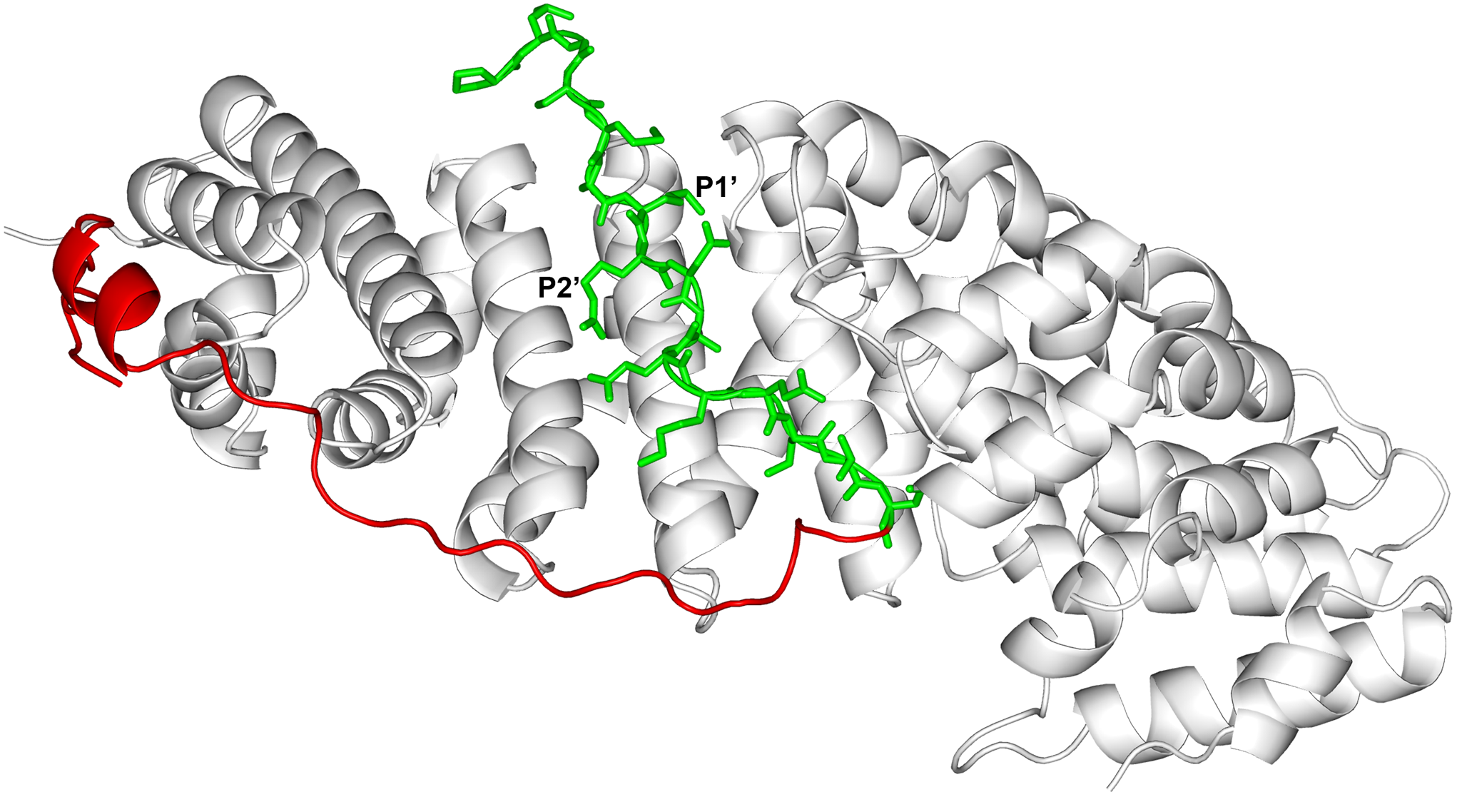

Supplement: Figure S1 — Structure of the Nup50-importin α complex (PDB entry 3TJ3). Importin α is shown in gray and Nup50 in green for residues −3 to 12 and red for the remainder. (TIF) [file pone.0091025.s001.tif]

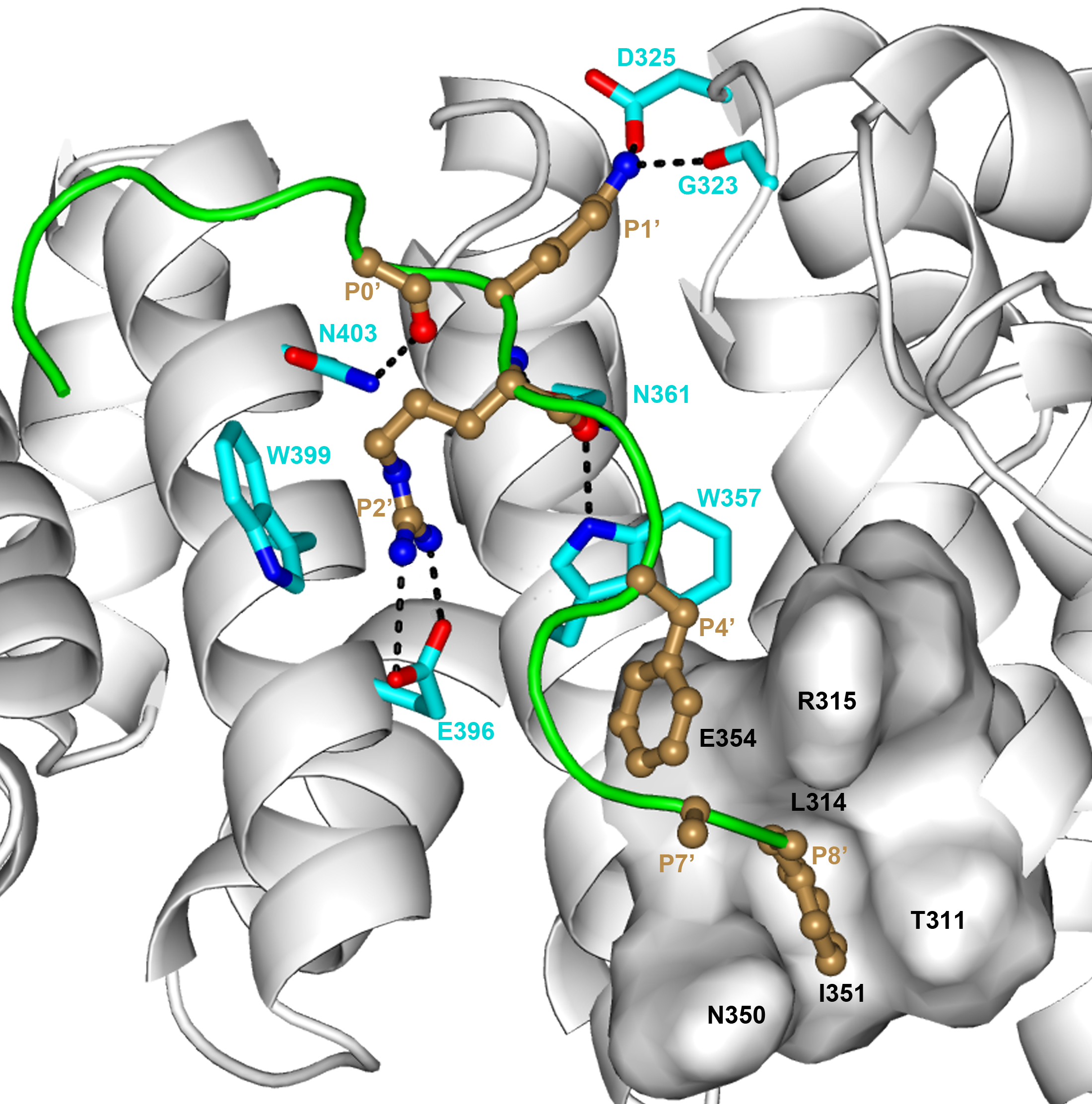

Supplement: Figure S2 — Interactions of NLS2 with the minor site of importin α. The color scheme is the same as in Figure 3. (TIF) [file pone.0091025.s002.tif]

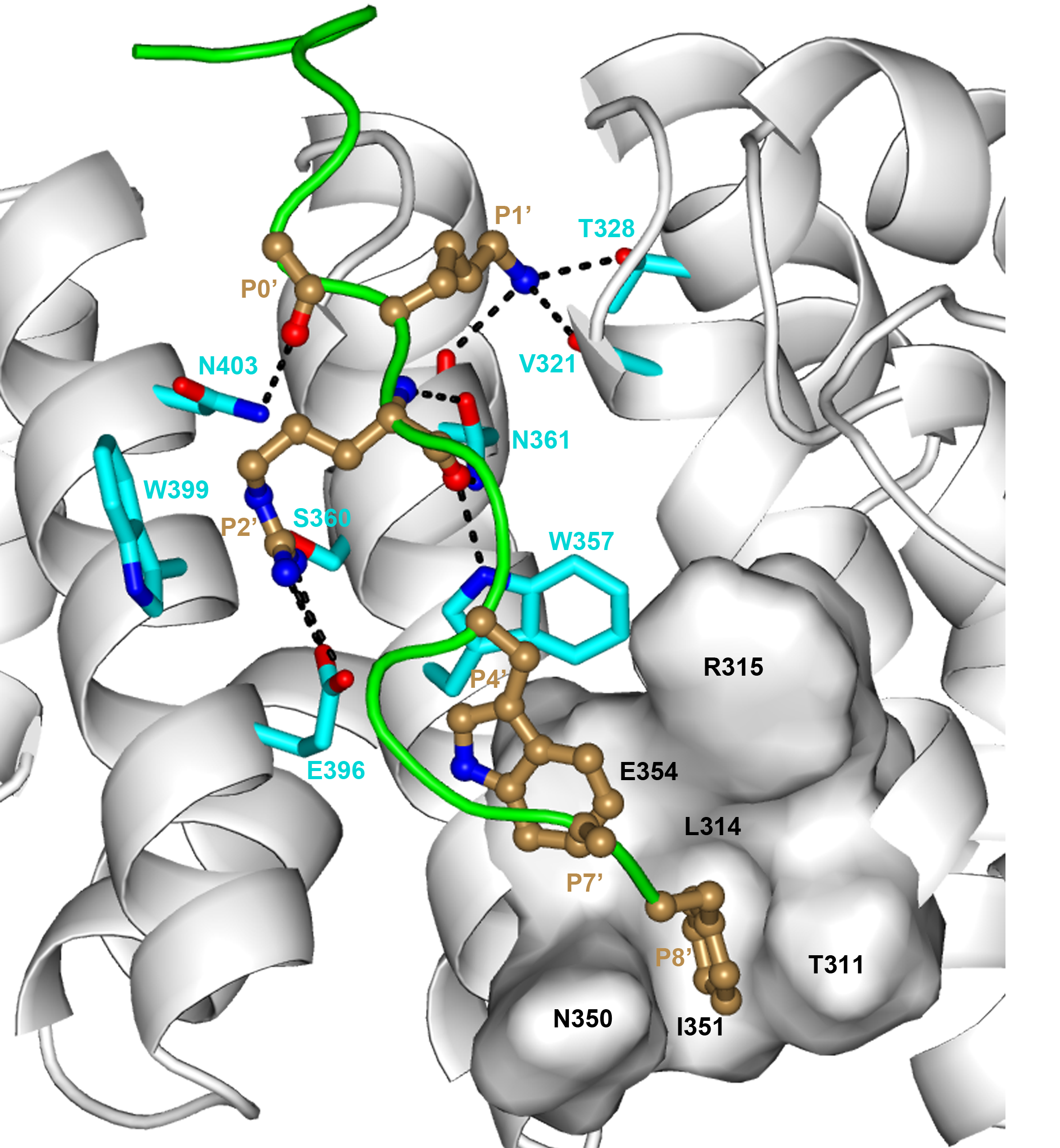

Supplement: Figure S3 — Interactions of NLS3 with the minor site of importin α. The color scheme is the same as in Figure 3. (TIF) [file pone.0091025.s003.tif]

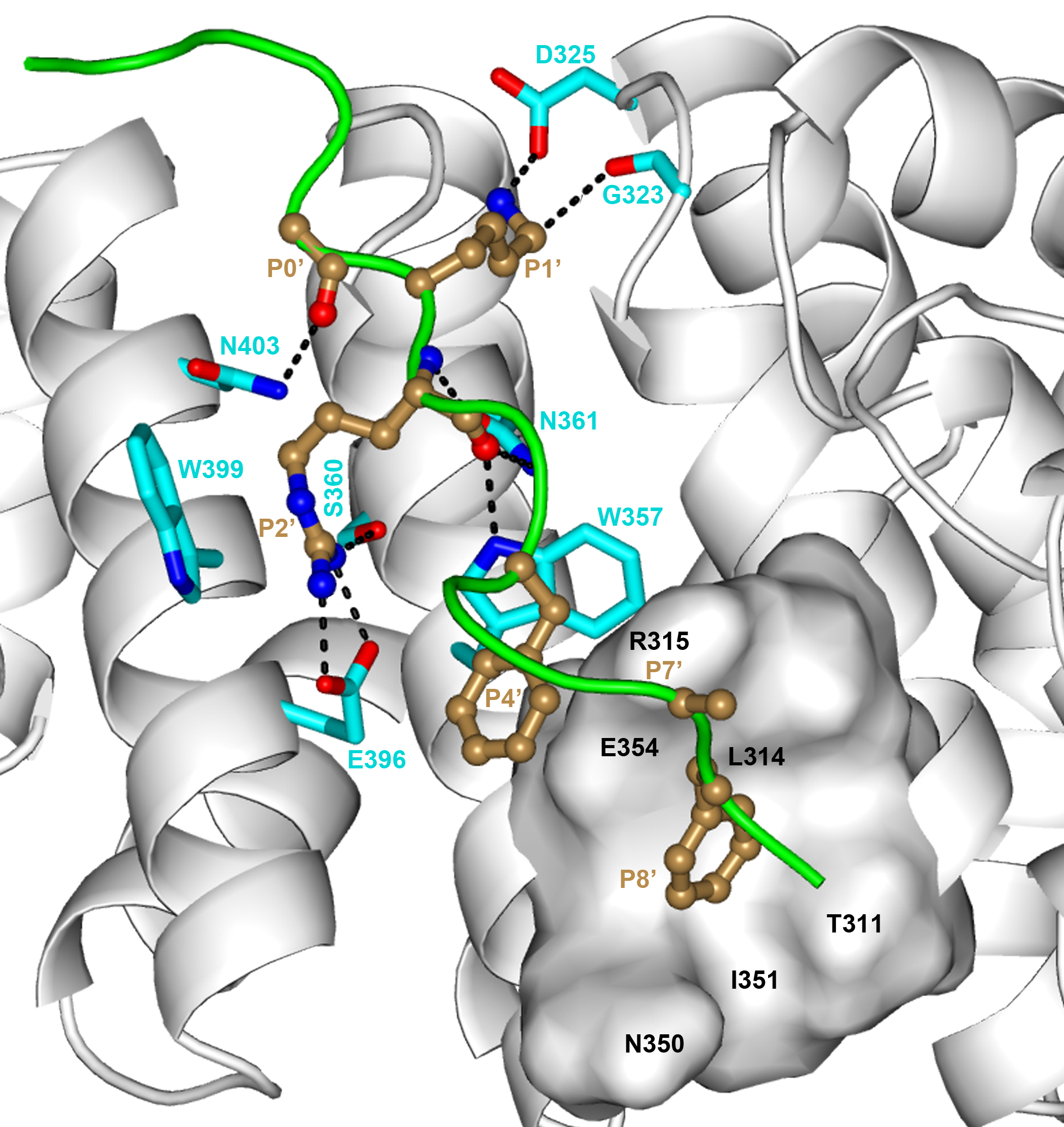

Supplement: Figure S4 — Interactions of NLS4 with the minor site of importin α. The color scheme is the same as in Figure 3. (TIF) [file pone.0091025.s004.tif]

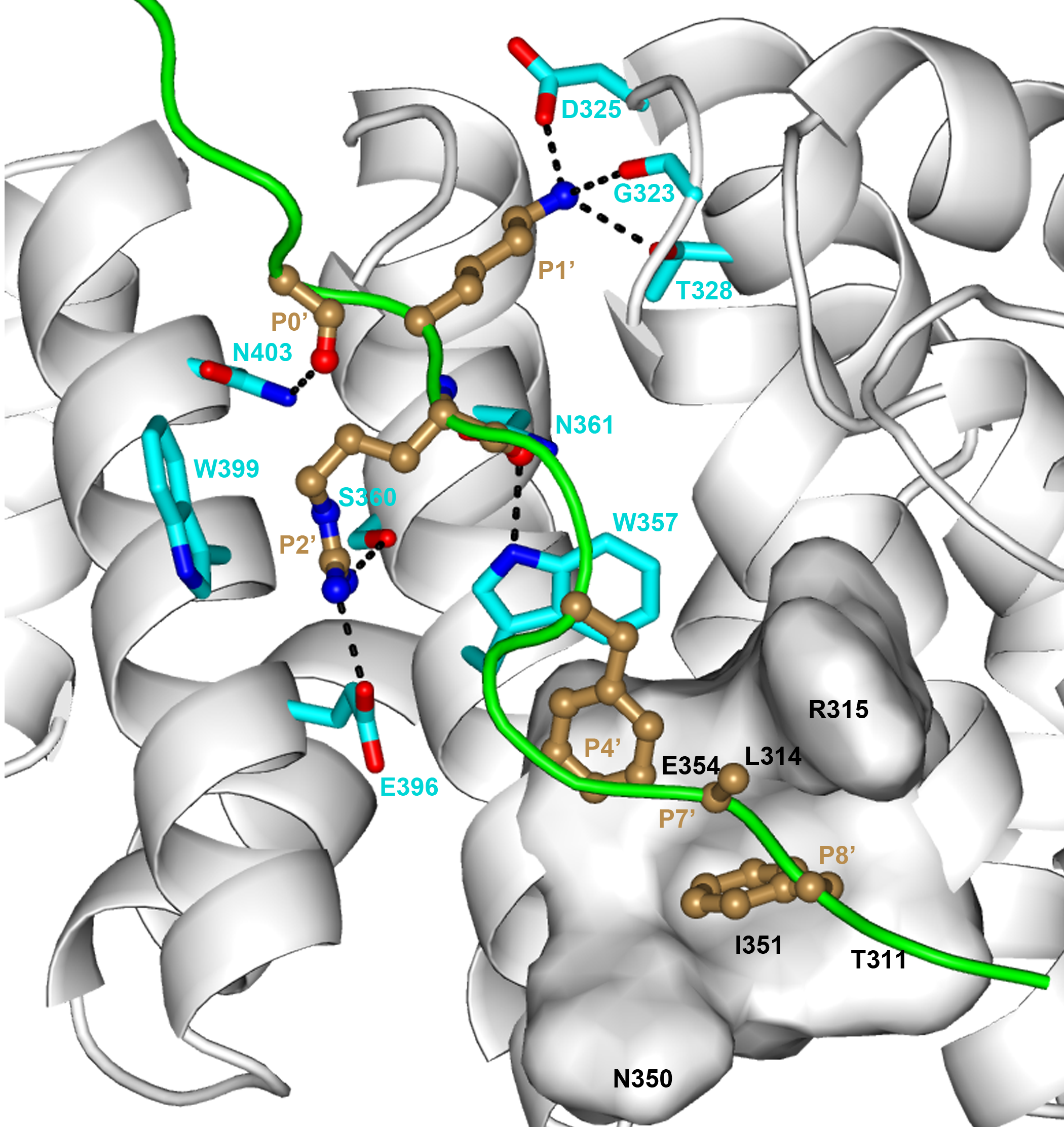

Supplement: Figure S5 — Interactions of NLS5 with the minor site of importin α. The color scheme is the same as in Figure 3. (TIF) [file pone.0091025.s005.tif]

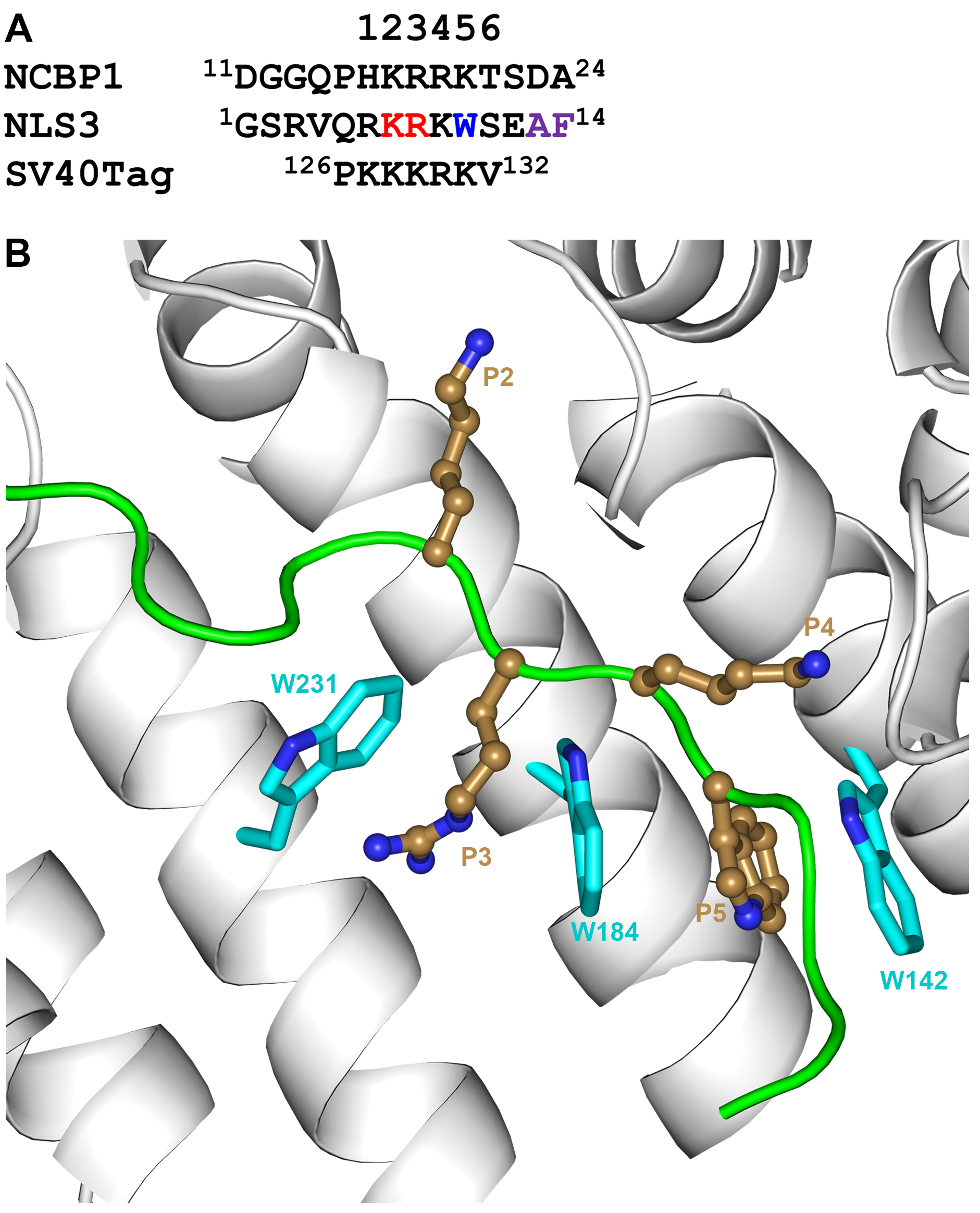

Supplement: Figure S6 — Model of NLS3 bound to the major site. (A) Sequence alignments of NLS3 and SV40Tag to NCBP1. The numbers at the top represent P positions. (B) The model for NLS3 bound to the major site of importin α, after refinement by Rosetta FlexPepDock and backbone-restrained MD simulation. The color scheme is the same as in Figure 3. (TIF) [file pone.0091025.s006.tif]

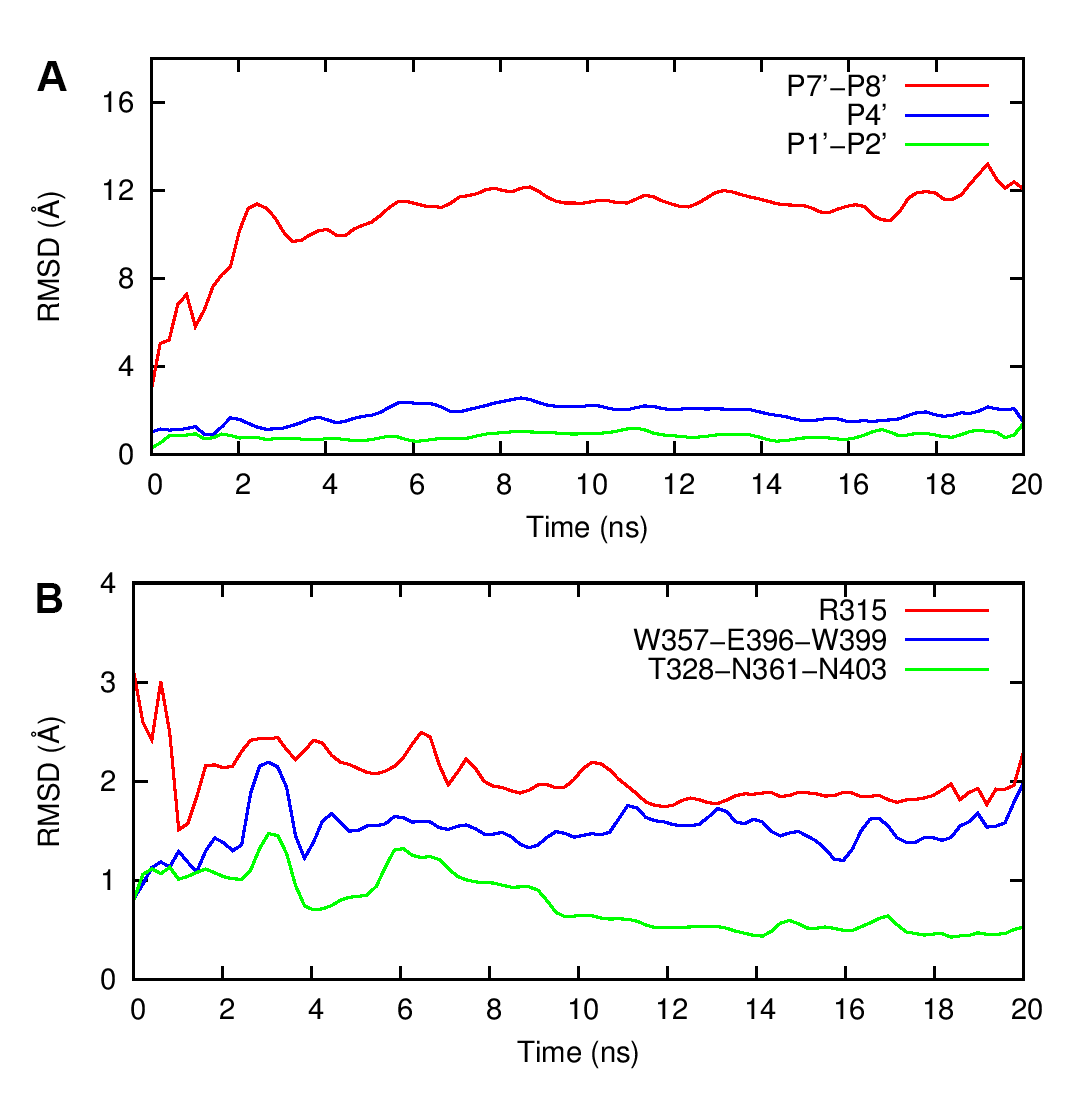

Supplement: Figure S7 — Relaxation of the minor-site bound model of NLS3 during an unrestrained MD simulation, as measured by RMSDs from the model. Superposition was done on the Cα atoms of importin α residues within 5 Å of NLS3. (A) Cα RMSDs of different parts of the peptide. (B) Sidechain tip atom RMSDs of protein residues that interact with the peptide. Tip atoms are: Cδ for E; Cγ for N; Cζ for R; Oγ1 for T; and Nε1 for W. (TIF) [file pone.0091025.s007.tif]

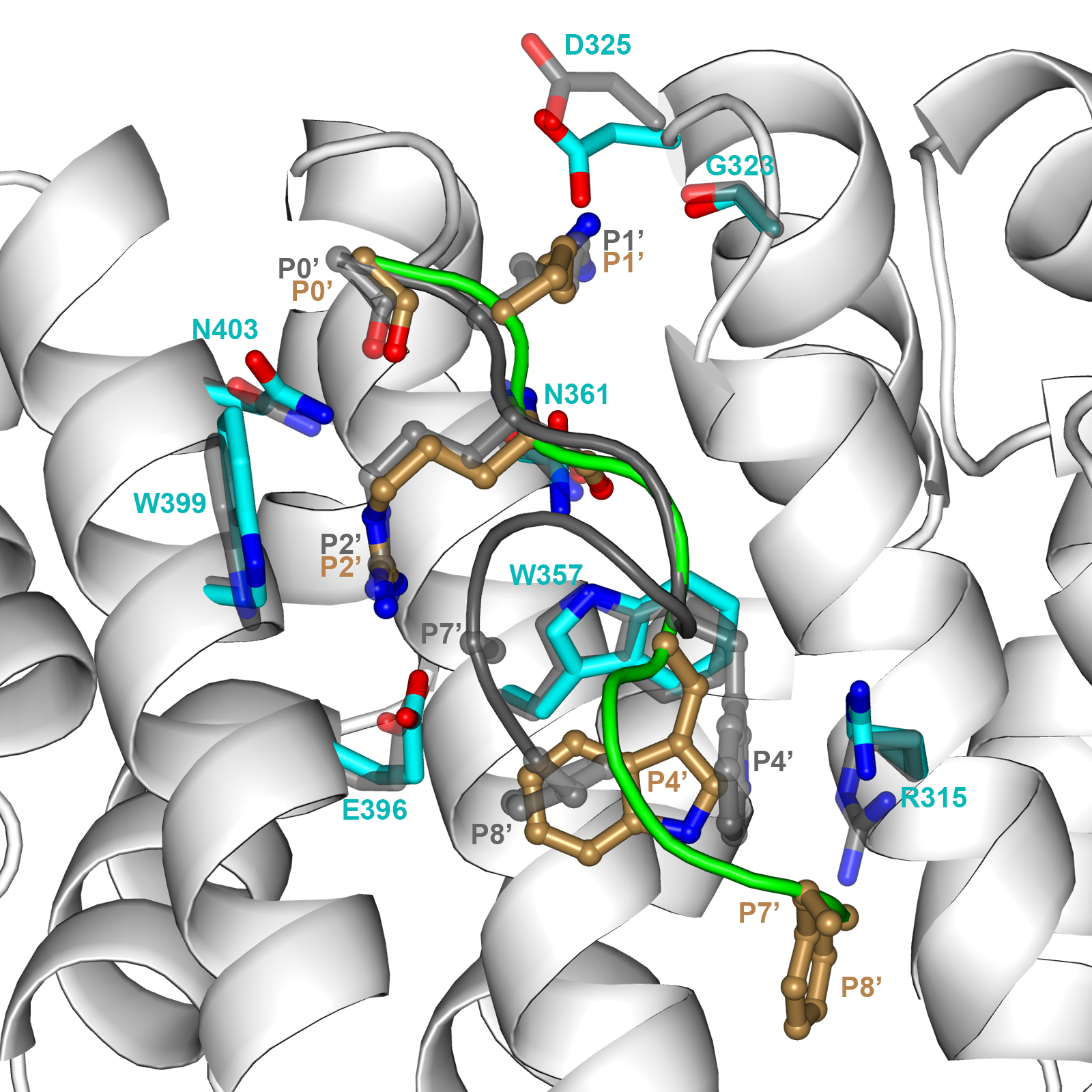

Supplement: Figure S8 — Comparison of the modeled and X-ray structures for the minor-site bound NLS1-importin α complex. The color scheme is the same as in Figure 5. (TIF) [file pone.0091025.s008.tif]

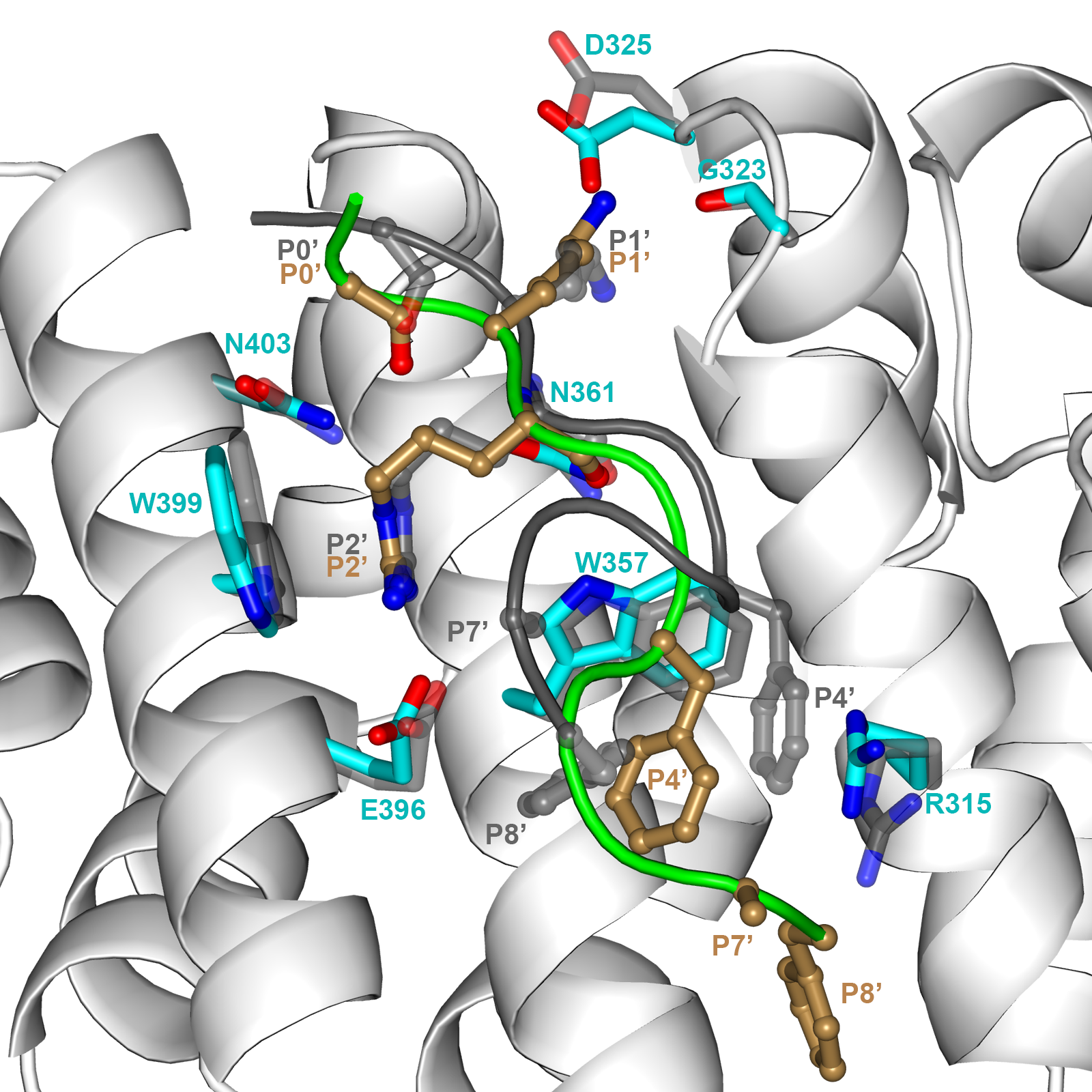

Supplement: Figure S9 — Comparison of the modeled and X-ray structures for the minor-site bound NLS2-importin α complex. The color scheme is the same as in Figure 5. (TIF) [file pone.0091025.s009.tif]

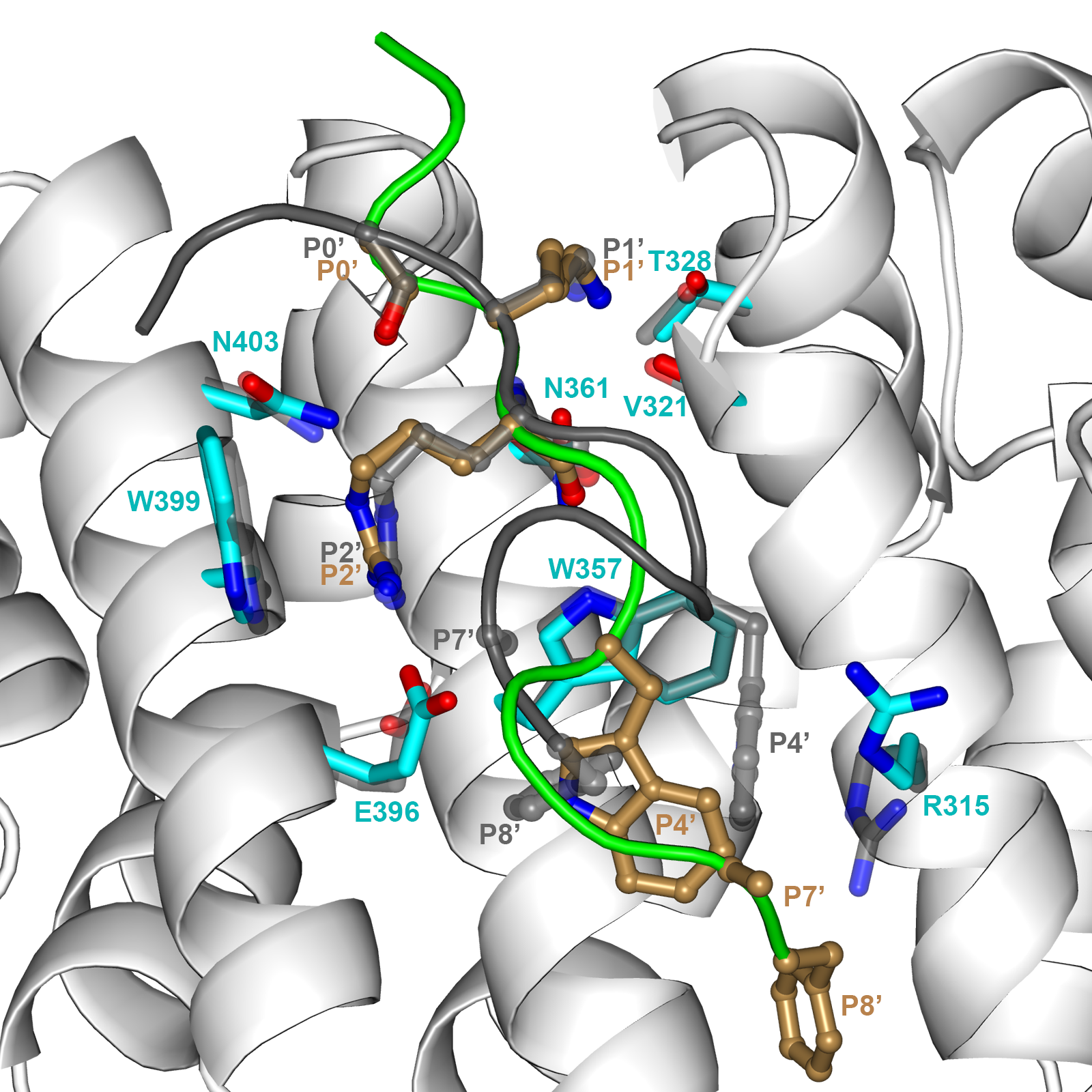

Supplement: Figure S10 — Comparison of the modeled and X-ray structures for the minor-site bound NLS3-importin α complex. The color scheme is the same as in Figure 5. (TIF) [file pone.0091025.s010.tif]

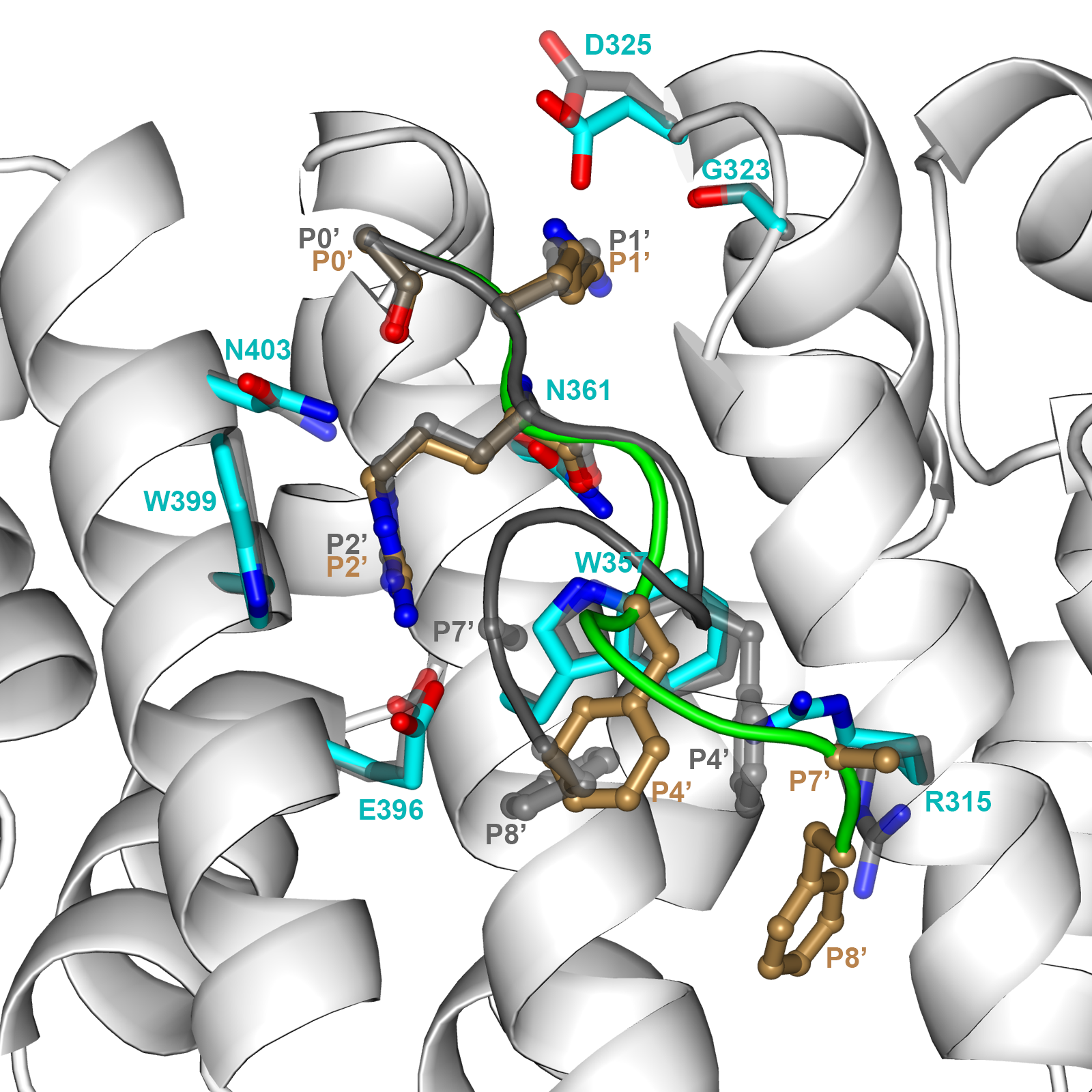

Supplement: Figure S11 — Comparison of the modeled and X-ray structures for the minor-site bound NLS4-importin α complex. The color scheme is the same as in Figure 5. (TIF) [file pone.0091025.s011.tif]

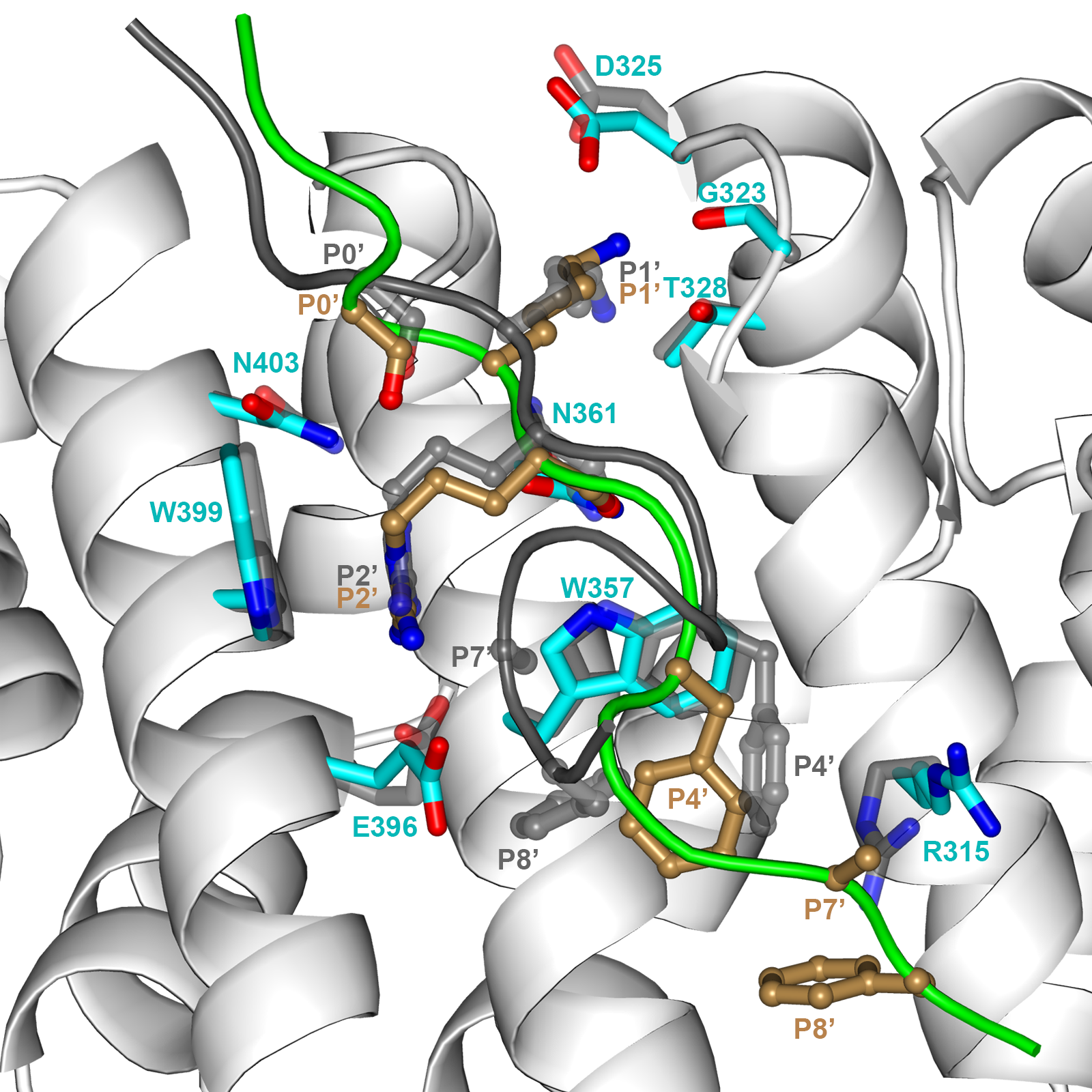

Supplement: Figure S12 — Comparison of the predicted and X-ray structures for the minor-site bound NLS5-importin α complex. The color scheme is the same as in Figure 5. (TIF) [file pone.0091025.s012.tif]

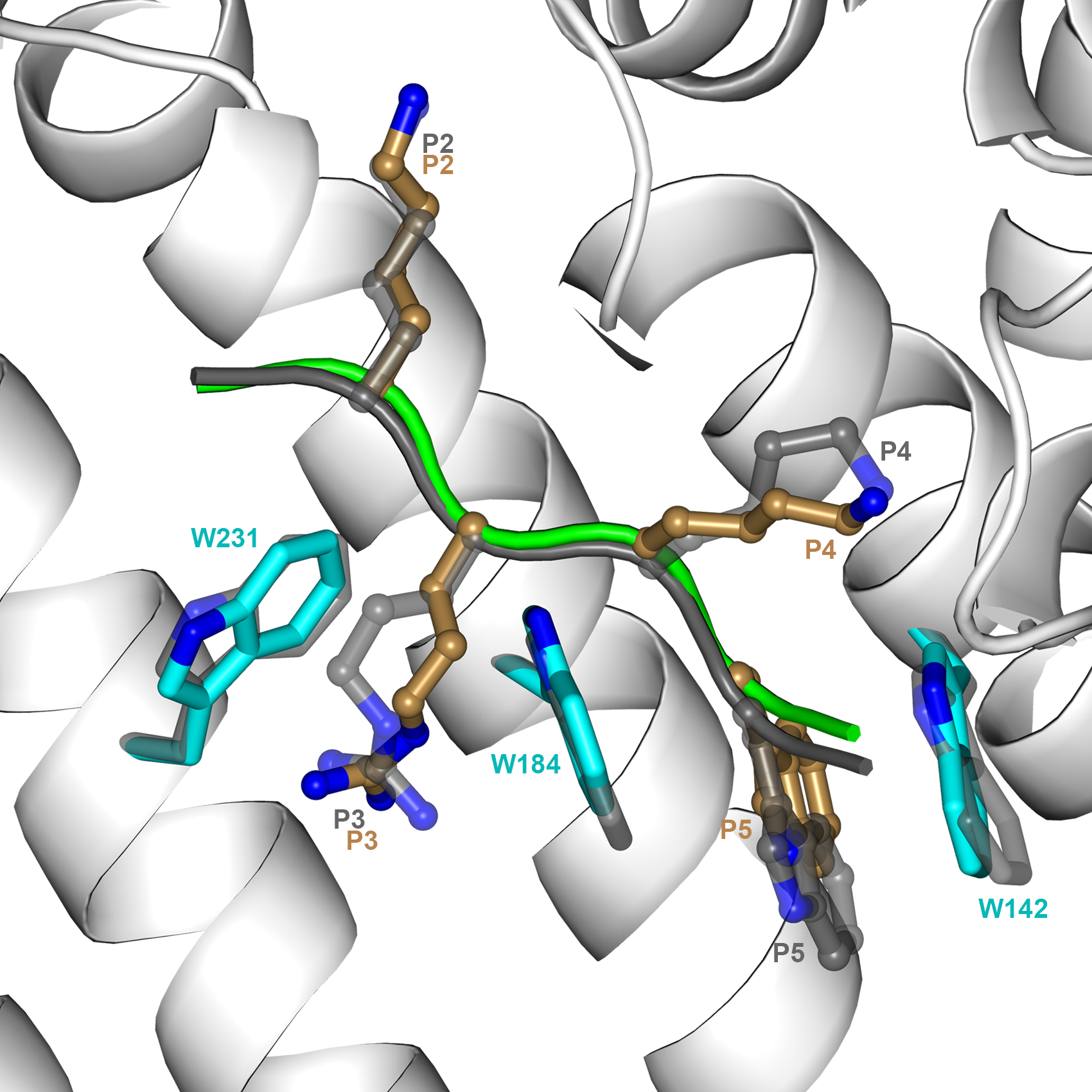

Supplement: Figure S13 — Comparison of the predicted and X-ray structures for the major-site bound NLS3-importin α complex. The color scheme is the same as in Figure 5. (TIF) [file pone.0091025.s013.tif]
