# Supplementary material for: Design Rules for Selective Binding of Nuclear Localization Signals to Minor Site of Importin α
Source: PLoS One. 2014 Mar 7;9(3):e91025. doi: 10.1371/journal.pone.0091025 (PMC3946659; doi:10.1371/journal.pone.0091025)
Supplement: Table S1 — Native proteins with sequences matching NLS1–5. (DOCX) [file pone.0091025.s014.docx]

**Table S1. Native proteins with sequences matching NLS1-5**

| **Sequence *^a^*** | **Protein Name** | **Organism** | **Length** | **UniPort ID** | **Function** |
| --- | --- | --- | --- | --- | --- |
| **^001^GSWAGRKRTWRDAF^14^** | NLS1 |  | 14 |  |  |
| **^001^GSSSHRKRKFSDAF^14^** | NLS2 |  | 14 |  |  |
| **^001^GSRVQRKRKWSEAF^14^** | NLS3 |  | 14 |  |  |
| **0^001^GSIGRKRGYSVAFG^14^** | NLS4 |  | 14 |  |  |
| **0^001^GSRGQKRSFSKAFGQ^15^** | NLS5 |  | 15 |  |  |
| **0^837^GNRGQKRSFSKAFGQ^851^** | Nucleolar RNA helicase II | Mus musculus | 851 | [Q9JIK5](http://www.uniprot.org/uniprot/Q9JIK5) | RNA helicase |
| **0^769^QNKGQKRSFSKAFGQ^783^** | Nucleolar RNA helicase II | Homo sapiens | 783 | [Q9NR30](http://www.uniprot.org/uniprot/Q9NR30) | RNA helicase |
| **^259^VVPASEKRKWSEAF^272^** | Methyl-CpG binding | Medicago truncatula | 286 | [Q2HU61](http://www.uniprot.org/uniprot/Q2HU61) | DNA binding |
| **^027^SVSLKRKRKFEDAF^40^** | BRCA1-A complex subunit RAP80 isoform 2 | Trichechus manatus latirostris | 719 | [XP_004371218.1](http://www.ncbi.nlm.nih.gov/protein/XP_004371218.1) | Ubiquitin binding |
| **0^393^FQIGYKRSFSKAFCS^407^** | Histone-lysine N-methyltransferase | Triticum urartu | 985 | [M7ZUV9](http://www.uniprot.org/uniprot/M7ZUV9) | Methyltransferase |

*^a^* The conserved KR, aromatic residue, and AF are in red, blue, and purple, respectively. Protein residues partially matching other positions of NLS1-5 are in green.
